# Concurrent jamming and mesenchymal-to-epithelial transitions during airway basal stem cell differentiation

**DOI:** 10.1016/j.isci.2026.115935

**Published:** 2026-04-28

**Authors:** Jennifer A. Mitchel, Jacob Notbohm, Reverie R. Brown, Mikoto A. Nakamura, Siddhant Kalra, Joseph D. Coolon, Chimwemwe Mwase, Michael J. O’Sullivan, Jin-Ah Park

**Affiliations:** 1 Department of Environmental Health, Harvard T.H. Chan School of Public Health, Boston, MA, USA; 2 Biology Department, Wesleyan University, Middletown, CT, USA; 3 Department of Mechanical Engineering, University of Wisconsin-Madison, Madison, WI, USA

**Keywords:** molecular biology, cell biology, stem cells research

## Abstract

Under homeostatic conditions, the human airway epithelium remains well-differentiated, stable, and resistant to external insults. This collective transitions between a stationary and a migratory state under various circumstances, including cellular differentiation and pathological conditions such as asthma. As human airway basal stem cells differentiate and form a stable tissue, they transition from a collectively migratory, fluid-like, unjammed phase to a stationary, solid-like, jammed phase. Here, we demonstrate that jamming during basal stem cell differentiation coincides with the mesenchymal-to-epithelial transition (MET). We show that cells from patients with asthma exhibit delayed MET and jamming, both of which are accelerated by the inhibition of TGF-β receptor activity. Reciprocally, TGF-β priming of healthy cells delays both MET and jamming, mimicking the delayed transitions observed in asthmatic cells. Our data reveal a central role for both epithelial-mesenchymal and jamming transitions in the cellular plasticity and dynamics governing the differentiation of human airway basal stem cells.

## Highlights

• Human airway stem cells undergo a jamming phase transition during differentiation
• The jamming transition (JT) coincides with the mesenchymal-to-epithelial transition
• JT and MET are delayed in airway stem cells cultured from asthmatic donors
• This delay is recapitulated or abrogated through the manipulation of TGF-βR activation

## Graphical abstract

**Figure:**
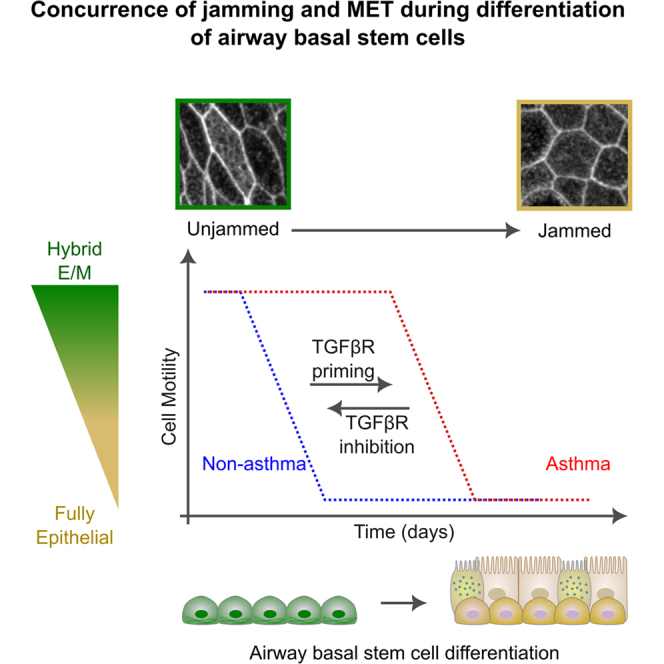

## Introduction

During embryonic development, cancer metastasis, and wound healing, the formation of new tissues requires rapid and reversible transitions between the stationary, adherent epithelial state and the migratory, detached mesenchymal state.^1^ This epithelial-mesenchymal plasticity (EMP) is driven by the evolutionarily conserved programs of the epithelial-to-mesenchymal transition (EMT) and its reverse, the mesenchymal-to-epithelial transition (MET).^2^ EMT initiates and propagates the deconstruction of apical-basal polarity and disassembly of stable intercellular junctions, whereas MET establishes apical-basal polarity and stable intercellular junctions.^3^ As a result, EMT produces mesenchymal cells while partial EMT produces hybrid cells with both epithelial and mesenchymal character (E/M cells), both of which can be highly migratory during development, cancer metastasis, and wound healing.^2^^,^^4^^,^^5^^,^^6^^,^^7^ By contrast, MET stabilizes migratory mesenchymal cells into stationary epithelial cells.^1^^,^^3^^,^^8^ For example, during development, MET programs generate various types of epithelia from developing germ layers, including the trophectoderm, endoderm, and mesoderm.^3^ During MET, individual cells with transient junctions migrate to their destination, where they become stationary and form a mature epithelium. Therefore, during development, MET plays a critical role in the stabilization and formation of the mature epithelium. However, MET may not be the sole program by which tissues transition from a migratory state to a stationary state, with recent evidence pointing to an important role for physical mechanisms such as the jamming transition (JT).^9^^,^^10^

In addition to the relatively well-studied processes involved in EMT and MET, recent work to understand multicellular plasticity has emphasized tissue-scale changes in cellular rheology and bulk material properties such as viscoelasticity.^11^^,^^12^^,^^13^^,^^14^ In various cellular collectives during dynamic processes, including the establishment of stable tissues,^15^ fluidization during morphogenesis,^10^^,^^12^^,^^16^^,^^17^^,^^18^ and the invasion of cancerous cells,^19^^,^^20^ the cellular collective transitions along a spectrum of apparent phases between solid-like and fluid-like. In the solid-like *jammed* phase, cells are caged by their neighbors and locked in place, while in the fluid-like *unjammed* phase, cells are uncaged from and swap places with their neighbors. During the transition from the jammed to the unjammed phase, termed the unjamming transition, cells can migrate, either through local neighbor swaps or, more often, cooperatively in spatially and temporally heterogeneous multicellular flocks.^9^^,^^10^^,^^21^^,^^22^^,^^23^^,^^24^ The unjamming transition is the process of loss of rigidity and concomitant gain of fluidity in a disordered system and occurs as initially jammed cellular collectives decrease in density due to loss of cell-cell contact or creation of a void^16^^,^^17^^,^^25^^,^^26^^,^^27^ or as cells increase their propulsion or active fluctuations.^15^^,^^21^^,^^28^^,^^29^^,^^30^ The unjamming transition can be triggered by a range of stimuli.^31^ *In vitro* experiments have demonstrated unjamming in response to radiation,^32^ increased endocytic recycling,^33^^,^^34^ changes in the microenvironment,^35^ and mechanical stretch or compression.^9^^,^^10^^,^^21^^,^^22^^,^^36^ *In vivo* experiments have highlighted the role of morphogen gradients,^37^ cell-cell contacts,^16^ and active cell movements^30^ in driving unjamming. By contrast, the JT is the process of emergence of rigidity in a disordered system.^14^^,^^38^^,^^39^ The JT occurs in cellular collectives in a variety of contexts, including as proliferating cells increase in density,^40^ as individualistic cancer cells are crowded into cooperativity by their confining extracellular environment,^19^^,^^20^ and as airway basal stem cells differentiate into a mature layer.^9^^,^^10^^,^^41^ These emerging lines of evidence together point to unjamming and JTs as physical mechanisms governing multicellular plasticity in a broad array of systems, but underlying molecular mechanisms are largely unknown.^31^ An important question that arises from these transitions is the extent to which the jamming and unjamming processes rely on the molecular programs that govern EMT and MET. Our previous work demonstrated that unjamming can occur independently of EMT,^21^ but the role of MET in jamming has not yet been determined.

Despite strong evidence implicating both MET and jamming in the solidification of a cellular collective and the corresponding cessation of movement, the relationship between these transitions remains largely undefined.^21^^,^^42^ Here, using maturing layers of primary airway basal stem cells isolated from human donors,^9^ we investigate the relationship between MET and the JT. During the maturation of airway epithelia, we evaluate the expression of canonical markers of epithelial and mesenchymal tissues in conjunction with the measurement of cellular motion and cell shape within the confluent cellular layer. We then hypothesize that the activation of TGF-β receptor (TGF-βR) contributes to both the observed hybrid E/M state and the degree of jamming in the maturing epithelial layer. We tested this hypothesis using a pharmacological inhibitor of TGF-βR I during layer maturation. We then further tested whether priming airway basal stem cells with exogenous TGF-β1 can alter either or both of MET or the JT. We observe that TGF-β1 priming recapitulates the delayed transitions and altered biophysical properties observed in primary airway basal stem cells cultured from human patients with asthma.^9^

## Results

### During differentiation of airway basal stem cells, MET, and jamming transitions occur simultaneously

**Figure 1:**
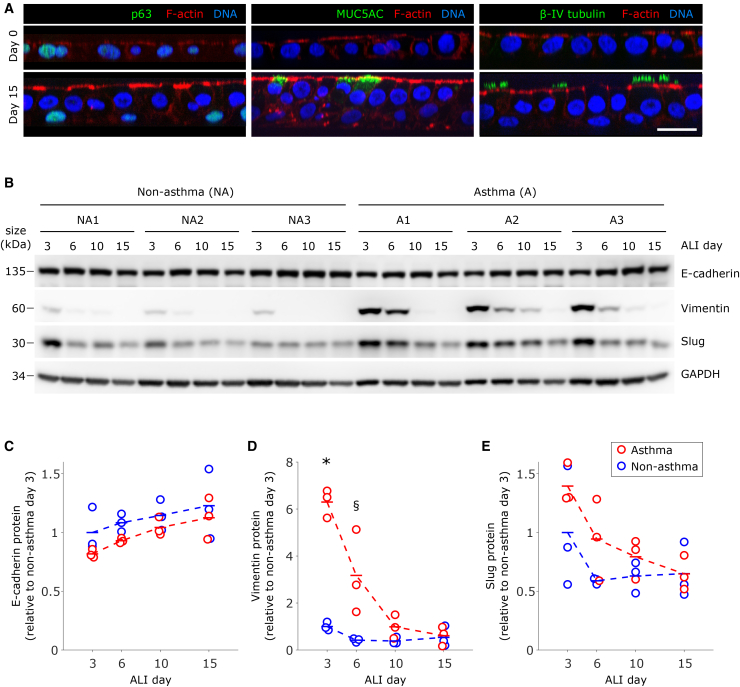
MET during the differentiation of airway epithelial cells Primary airway basal stem cells are cultured at the air-liquid interface (ALI). (A) Cell composition is shown by immunofluorescence staining of p63, MUC5AC, and β-IV tubulin, which mark basal stem cells, goblet cells, and ciliated cells, respectively (scale bars are 20 μm). On ALI day 0, only basal stem cells are present, as indicated by p63 staining and the absence of differentiation markers MUC5AC and β-IV tubulin (A, top). By ALI day 15, the tissue has become pseudostratified and contains p63-positive basal stem cells, MUC5AC-positive goblet cells, and β-IV tubulin-positive ciliated cells (A, bottom). Expression of epithelial marker E-cadherin and mesenchymal markers vimentin and slug was determined by western blot in cells isolated from non-asthmatic (*n* = 3) and asthmatic (*n* = 3) donors cultured at the air-liquid interface (ALI) on days 3, 6, 10, and 15, with a representative western blot shown in (B). Quantification was performed by densitometry analysis (C–E), and each symbol represents the protein levels for a single donor, averaged across two biological replicates. (C) Expression of epithelial marker E-cadherin shows a trend of increasing levels over the course of differentiation. (D) Expression of mesenchymal marker vimentin decreased over the course of differentiation in cells from both normal and asthmatic donors. In asthmatic cells, compared to non-asthmatic cells, vimentin levels were significantly higher on ALI day 3 (∗*p* < 0.001, *t* test non-asthmatic vs. asthmatic) and trended toward being higher on ALI day 6 (§*p* = 0.055). (E) Expression of EMT-TF slug was detectable on all ALI days in cells from both non-asthma and asthma donors and showed a trend toward decreasing over time in both cell types. (C–E) Blue symbols correspond to non-asthmatic donors, while red symbols correspond to asthmatic donors.

When grown in an air-liquid interface (ALI) culture, primary human airway basal stem cells, which are conventionally called human bronchial epithelial (HBE) cells, mature and differentiate into a pseudostratified epithelium that recapitulates the *in vivo* human bronchial airway epithelium.^43^^,^^44^^,^^45^ *In vitro* culture of HBE cells is initiated in a submerged condition, when basal cells are plated on porous transwell inserts, where the cellular layer initially comprises airway basal stem cells solely. When the cellular layer reaches confluency, the culture medium is then removed from the apical side of the cellular layer, where the cellular layer is now established for ALI culture. On the day when ALI culture is established, which is marked as ALI day 0, the cellular layer still comprises only basal cells (Figure 1A).^46^ After cells are maintained in ALI culture for 15 days, which is denoted as ALI day 15, the cellular layer now comprises basal cells as well as various differentiated airway epithelial cells, including goblet and ciliated cells (Figure 1A).^44^^,^^45^^,^^47^^,^^48^ During the differentiation of the HBE cells in ALI culture, the confluent cellular layer undergoes a progressive phase transition from a fluid-like, unjammed phase on early ALI days to a solid-like, jammed phase on later ALI days.^9^^,^^10^ This transition from the unjammed to the jammed phase is substantially delayed when the primary cells are cultured from patients with lung diseases, including idiopathic pulmonary fibrosis (IPF), COPD, or asthma.^9^^,^^10^^,^^41^^,^^49^ Though the JT in this system occurs in parallel with maturation and differentiation, the underlying molecular mechanisms of the JT in this system are unknown.^9^^,^^31^^,^^39^^,^^42^

Because MET is a well-known mechanism for the maturation of epithelial tissues from migratory precursors, we first examined the expression of proteins that play important roles in EMT and MET.^50^ We first detected the cellular expression of the epithelial marker E-cadherin along with the well-known mesenchymal marker vimentin^21^^,^^51^ and an EMT-inducing transcription factor (EMT-TF) slug (Snai2)^50^^,^^52^ (Figure 1). To examine changes in the expression of these proteins in HBE cells from donors with no history of lung disease (denoted as non-asthma, *n* = 3 donors), we performed western blot analysis on cell lysates collected on ALI days 3, 6, 10, and 15 (Figure 1B). From ALI day 3–15, the expression of the epithelial cell marker, e-cadherin, appeared to be abundant with a slight increase throughout ALI days (Figures 1B and 1C). Vimentin was readily detectable only on ALI day 3 and became barely detectable or undetectable on ALI days 6, 10, and 15 (Figures 1B–1D). Slug was most prominent on ALI day 3, but unlike vimentin, it remained detectable through ALI day 15 (Figures 1B–1E). These data indicate that at an early ALI day, HBE cell layers exhibit a hybrid E/M phenotype, but during differentiation undergo a transition from partially mesenchymal toward fully mature epithelium, consistent with MET.

We then determined whether this transition from a hybrid E/M state to a mature epithelial state takes place also in HBE cells from donors with asthma. In patients with asthma, the airway epithelium is dysregulated, which is increasingly recognized as a core driver of disease progression.^53^^,^^54^^,^^55^^,^^56^ When grown in ALI culture, HBE cell layers cultured from patients with asthma recapitulate aspects of the pathologically remodeled airways, including an increased number of goblet cells,^57^ impaired antiviral function,^58^^,^^59^^,^^60^ increased susceptibility to allergens,^61^ disrupted barrier function,^62^^,^^63^ and delayed maturation.^9^^,^^58^ As with non-asthmatic HBE cells, in cell culture from patients with asthma (denoted as asthma, *n* = 3 donors), we detected vimentin, slug, and E-cadherin on ALI days 3, 6, 10, and 15. In these asthmatic epithelia, the level of vimentin was significantly higher than that in non-asthmatic epithelia on ALI day 3 and continued to decline toward ALI day 15. The level of slug followed a similar trend (Figures 1B–1E). In asthmatic epithelial cells, the pattern of temporal reduction of vimentin and slug contrasted with that in non-asthmatic epithelia, where the levels of vimentin and slug reached a relatively low or undetectable steady state by ALI day 6 and remained through ALI day 15 (Figures 1B–1E). As in non-asthmatic cells, the cellular expression of E-cadherin appeared to be abundant, with a slight increase throughout ALI days (Figures 1B and 1C). In asthmatic cells, as in non-asthmatic cells, the observed reductions of vimentin and slug over the course of ALI days, concomitant with the strong expression of E-cadherin, suggest that during differentiation asthmatic cells may also undergo MET. However, this MET and concomitant loss of the hybrid E/M phenotype is relatively *delayed* in asthmatic HBE cell layers (Figures 1B–1E). This delay in MET mirrors the delay in the JT previously observed in asthmatic cells.^9^^,^^10^

### TGF-β receptor signaling is a key regulator of both MET and jamming transitions

**Figure 2:**
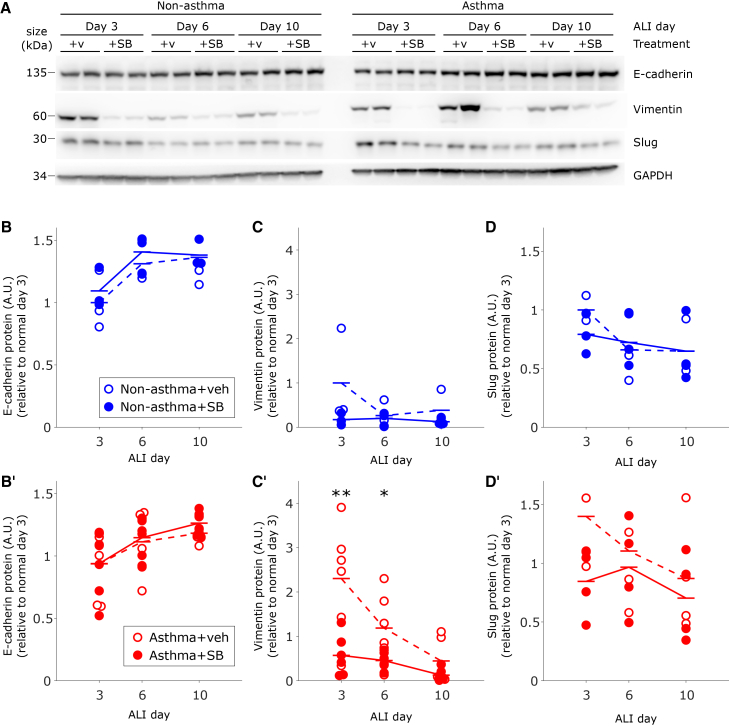
Blocking TGFβR activity accelerates MET in asthmatic epithelium HBE cells were collected on ALI days 3, 6, and 10, after 3 days of treatment with vehicle control or TGFβR inhibitor (SB-431541 (SB), 5 μM). (A) Expression of epithelial marker e-cadherin and mesenchymal markers vimentin and slug was determined by western blot in cells isolated from non-asthmatic (*n* = 3) and asthmatic (*n* = 6) donors. Quantification was performed by densitometry analysis (B–D), and each symbol represents the protein levels for a single donor, averaged across two biological replicates. Expression of epithelial marker E-cadherin shows a trend of increasing levels over the course of differentiation and was unaffected by treatment with SB in either non-asthmatic (B) or asthmatic (B′) cells. In non-asthmatic cells, vimentin expression was overall relatively low and unaffected by treatment with SB (C), while in asthmatic cells, vimentin expression was significantly reduced by treatment with SB on ALI days 3 and 6 (C′, ∗∗*p* < 0.01 and ∗*p* < 0.05, *t* test vehicle-vs. SB-treated). In both non-asthmatic and asthmatic cells, slug expression exhibited a trend toward decreasing over time and was unaffected by treatment with SB (D, D'). (B–D) Open symbols denote vehicle treatment, while closed symbols denote SB treatment; blue symbols correspond to non-asthmatic donors, while red symbols correspond to asthmatic donors.

To interrogate underlying intracellular signaling pathways that regulate MET during differentiation, we focused on the role of TGF-βR, the activation of which is tightly regulated in epithelial cell proliferation and differentiation^64^ and which is known to play a critical role in the fate of epithelial progenitor cells.^65^^,^^66^^,^^67^ In our RNA-seq analysis from HBE cells collected on ALI days 0, 1, 7, and 14, we detected that the expression of genes involved in the activation of TGF-βR, including *TGFB1*, *TGFBR1*, *TGFB2*, and *TGFBR2*, is all progressively decreased during differentiation in ALI culture (Figure S1). We therefore hypothesize that TGF-βR signaling is decreasing concurrently with the JT in this system. Furthermore, during EMT, the activation of TGF-βR induces the expression of vimentin and slug,^21^^,^^68^ the expression of which progressively decreased during the differentiation of HBE cells in ALI culture (Figures 1 and S1). To test if TGF-βR controls the kinetics of MET in this system, we blocked TGF-βR activity using a specific pharmacological inhibitor, SB-431542 (denoted as SB, used at 5 μM) during differentiation. In ALI culture, cells were incubated with SB for 3 days prior to each of the three time points where we examined epithelial and mesenchymal markers: ALI days 3, 6, and 10 (Figure 2).

For HBE cell layers cultured from both non-asthmatic and asthmatic donors, treatment with SB did not affect the level of E-cadherin or slug expression (Figures 2A, 2B, 2B′, 2D, and 2D′). For non-asthmatic cells (*n* = 3 donors), TGF-βR inhibition did not significantly affect the protein expression of the mesenchymal markers, though there was a trend toward the reduction of vimentin on ALI day 3 (Figures 2A–2C). By contrast, for asthmatic cells (*n* = 6 donors), TGF-βR inhibition significantly reduced vimentin expression on both ALI days 3 and 6, with a trend toward reduced expression on ALI day 10 (Figures 2A–2C′). Importantly, TGF-βR inhibition in asthmatic cells reduced vimentin expression to similar levels as observed in non-asthmatic cells, indicating that this treatment could rescue the delayed MET observed in asthmatic cells.

**Figure 3:**
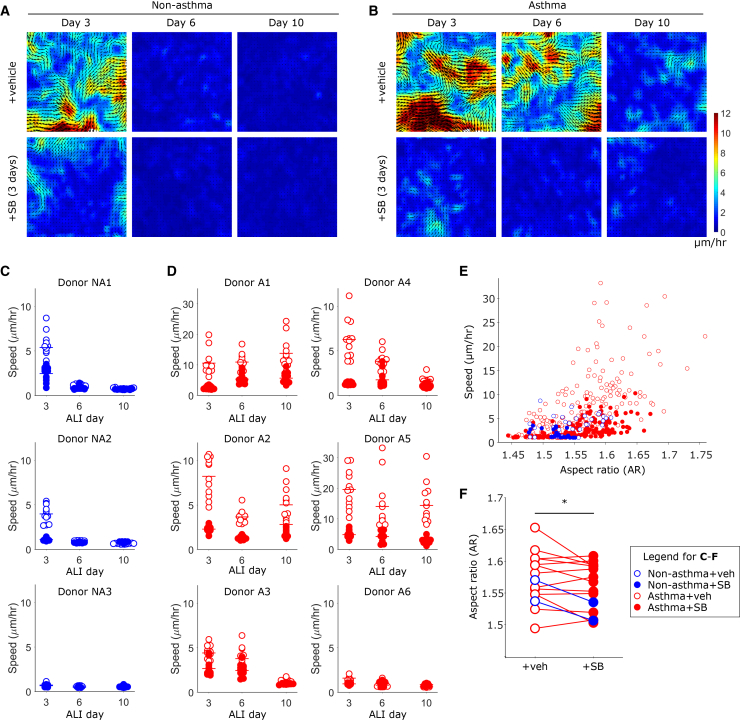
Blocking TGFβR activity accelerates jamming transition in asthmatic epithelium HBE cells were imaged to determine dynamics on ALI days 3, 6, and 10, after 3 days of treatment with vehicle control or TGFβR inhibitor (SB-431541 (SB), 5 μM). Representative speed maps for r non-asthmatic (A, donor NA2) and asthmatic (B, donor A4) HBE cells show that the jamming transition was relatively delayed in asthmatic donors, and that treatment with SB abrogated cellular migration. Quantification of average speed in each field of view for *n* = 3 non-asthmatic donors (C) and *n* = 6 asthmatic donors (D) shows the high degree of variability between donors. Note that the y axis scale is substantially increased for Donors A1 and A5. (E) Cell shape elongation, as measured by aspect ratio (AR), is significantly positively correlated with cell speed (*p* < 0.0001, r = 0.7680, Spearman’s correlation), such that faster moving cells have a higher AR across all conditions. Each symbol corresponds to the average cell shape and average speed measured from a single field of view. (F) Inhibition of TGFβR signaling reduced cell elongation. Conditions where HBE cells were migratory under vehicle treatment were determined; under these conditions of baseline unjammed behavior, treatment with SB significantly reduced AR (*p* < 0.05, paired *t* test). Each symbol corresponds to an average across 12 fields of view. (C-F) Open symbols denote vehicle treatment, while closed symbols denote SB treatment; blue symbols correspond to non-asthmatic donors, while red symbols correspond to asthmatic donors.

Because the TGF-βR-inhibition accelerated the MET in asthmatic cells, we examined whether the same treatment could accelerate the JT. Using the same conditions described for Figure 2, we analyzed cellular migration and cell shape to determine the jamming phase (Figure 3). We display data for each of the 3 non-diseased and 6 asthmatic donors individually to allow the visualization of the heterogeneity between donors. As expected, in cell layers cultured from non-asthmatic donors treated with vehicle, the JT occurred relatively early, before ALI day 6 (Figures 3A–3C). Of the three non-asthmatic donors evaluated, one was jammed even before ALI day 3, while the other two remained unjammed on ALI day 3 (Figure 3C, donors NA1 and NA2). For these two donors, TGF-βR inhibition (SB treated during days 0–3) reduced the speed of cellular migration on ALI day 3. In all three non-asthmatic donors, TGF-βR inhibition showed no noticeable difference in cellular migration on ALI days 6 and 10, as the layers were already jammed.^9^^,^^10^

In cell layers cultured from asthmatic donors treated with vehicle, the onset of the JT was highly variable between donors, ranging from day 6 to after day 10, in agreement with previous reports^9^^,^^10^ (Figures 3B–3D). In one of the six asthmatic donors, cell layers jammed by day 6, similarly to the non-asthmatic donors; here, TGF-βR inhibition showed no noticeable difference in cellular migration (Figure 3D, donor A6). In the remaining five asthmatic donors (donors A1-A5), TGF-βR inhibition reduced cellular migration on all days where the untreated cells otherwise remained unjammed. In particular, in the three asthmatic donors where vehicle-treated cells were still substantially migrating on ALI day 10, TGF-βR inhibition rescued delayed jamming (Figure 3D, donors A1, A2, A5).

Previous theoretical and experimental work has demonstrated tight correspondence between cell shape and cell dynamics during jamming and unjamming phase transitions, such that cells that rearrange less frequently and are less migratory are more rounded in shape, compared to cells that are more migratory, which in turn exhibit a more elongated and variable morphology.^9^^,^^10^^,^^21^^,^^24^^,^^33^^,^^36^^,^^69^^,^^70^^,^^71^^,^^72^ Current evidence suggests that this relationship is determined by the balance of forces between neighboring cells, with contributions from cortical tension, cell-cell adhesion, cell-generated motility forces, and in some cases, tissue-scale anisotropy.^15^^,^^24^^,^^29^^,^^33^^,^^72^^,^^73^ Therefore, using a machine learning algorithm trained on phase contrast images from timelapse movies of cell dynamics, we measured cell shapes by determining the aspect ratio (AR) of HBE cells across our experiments. As in previous studies, we find that there is a significant correlation between AR and cellular migration speed, such that faster moving cells are more elongated (*p* < 0.0001, r = 0.7680, Figure 3E). Furthermore, for conditions in which vehicle-treated cells were migratory (defined as an average migration speed of 1.8 μm/h or greater), treatment with SB significantly reduced the AR (*p* < 0.05, Figure 3F). This data supports our understanding that TGF-βR inhibition promotes a JT in unjammed epithelia.

Last, because the inhibition of TGF-βR reduced both vimentin expression (Figures 2A–2C) and cellular migration speed (Figures 3A–3D), we examined the correlative relationship between these two outcomes. We compared the relative level of vimentin expressed to the average speed of the cells on a well-by-well basis. Overall, there is a significant correlation between vimentin expression and speed (*p* < 0.0001, r = 0.53). For conditions in which vimentin expression is relatively high, vimentin expression significantly correlates with cell speed (Figure S2). However, because we observed certain conditions (three of six asthmatic donors on ALI day 10) where the epithelial layers were highly migratory (with average cell speeds >5 μm/h) but expressed low baseline levels of vimentin (Figure S2A), we conclude that that vimentin is not necessary for migration.

Together, our data indicate that, in this system of primary HBE cells differentiating in ALI culture, cells from both non-diseased and asthmatic donors undergo concurrent MET and JTs, and that both transitions can be accelerated by the inhibition of TGF-βR-signaling (Figures 2 and 3).

### Priming airway basal stem cells with TGF-β recapitulates delayed phase transitions of asthmatic cells

Because blocking of TGF-βR accelerated the delayed phase transitions of the asthmatic cells, we further hypothesize that delayed phase transitions are established by the activation of TGF-βR signaling. However, because the direct activation of TGF-βR through treatment with TGF-β in HBE cells during ALI culture promotes EMT,^21^^,^^74^ we instead adapted the approach of preconditioning, or priming, a technique that has been widely used in stem cell-based therapies. Priming stem cells with cytokines or by specific culture conditions alters their differentiation potential.^3^^,^^75^^,^^76^^,^^77^ Here we tested if priming of non-asthmatic basal stem cells with TGF-β1 at a low concentration, one log order lower than the concentration inducing EMT, could shift the phenotype of non-asthmatic cells toward asthmatic cells, even in the absence of continued TGF-β1 stimulation during differentiation. Importantly, TGF-β1 is elevated in the bronchoalveolar lavage of patients with asthma,^78^ and, therefore, may be an important factor continuously priming basal cells in the asthmatic airway. Thus, priming non-diseased HBE cells may mimic the exposure of cells in patient airways to TGF-β1.

As is standard in the propagation of primary HBE cells, we expanded the airway stem cells from non-diseased donors from passage 1 to passage 2 in flasks prior to culture in transwells.^43^^,^^79^ To generate *primed* HBE cells, we incubated basal stem cells cultured from non-diseased donors with low dose (0.3 ng/mL) TGF-β1, or vehicle control, for 2–3 days during this expansion. This low dose was chosen in contrast to doses used in previously published studies, which sought to induce full or partial EMT. For example, in our own published work using well-differentiated HBE cells, we induce partial EMT with 10 ng/mL of TGF-β1 for 3 days.^21^ Other groups have used 3–30 ng/mL of TGF-β1 for up to two weeks to induce full EMT in other epithelial models, including A549 lung adenocarcinoma, MCF10A mammary breast epithelium, and murine mammary epithelial cells.^80^^,^^81^^,^^82^ These passages 2 cells (both primed and unprimed controls) were then harvested and plated on transwells to differentiate in ALI culture using standard techniques. On ALI days 3, 6, 10, and 14, we examined canonical markers of MET and jamming.

**Figure 4:**
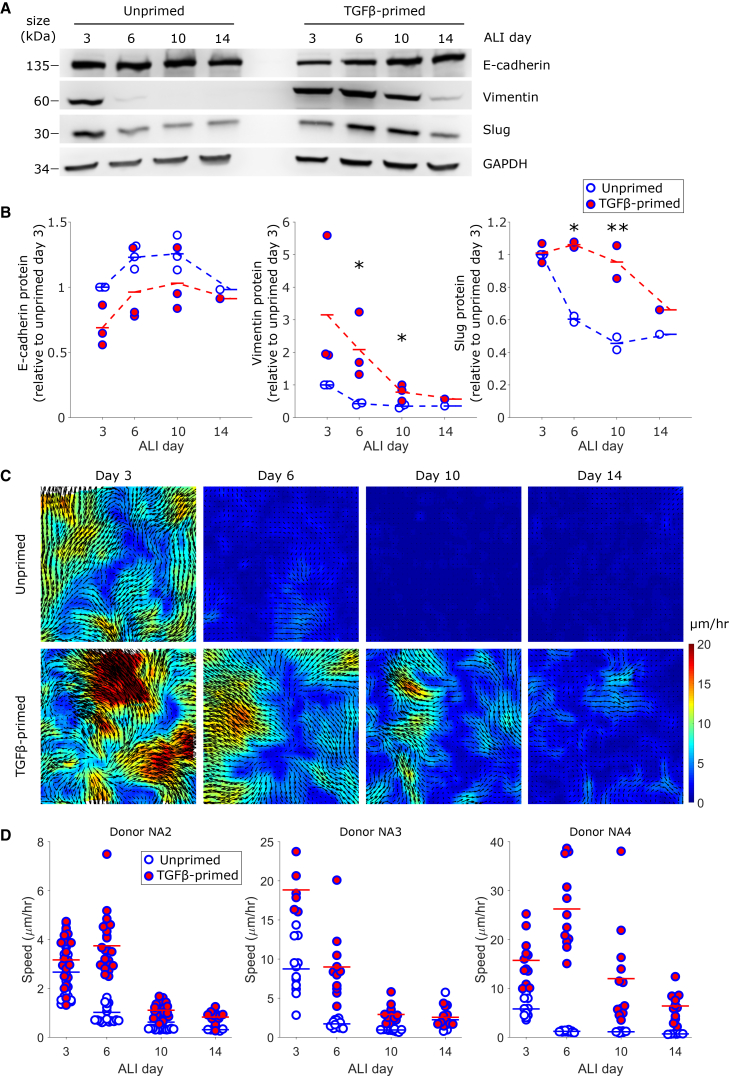
Healthy airway stem cells primed with TGFβ phenocopy the delayed jamming transition of asthmatic cells Unprimed control and TGFβ-primed cells were generated from *n* = 3 non-asthmatic donors and differentiated in ALI. Cells were evaluated for their protein expression (A, B) and dynamics (C, D) on ALI days 3, 6, 10, and 14. (A) Representative Western blot shows the protein expression of epithelial marker E-cadherin and mesenchymal markers vimentin and slug. The pattern of expression of these epithelial and mesenchymal markers in the primed cells mimics that observed in asthmatic cells. Quantification was performed by densitometry analysis (B), and each symbol represents the protein levels for a single donor, averaged across two biological replicates. (B) Expression of epithelial marker E-cadherin shows a trend of increasing levels over the course of differentiation. Expression of mesenchymal marker vimentin decreased over the course of differentiation in cells from both control and primed cells. In primed cells, compared to control cells, vimentin levels were significantly higher on ALI days 6 and 10 (∗*p* < 0.05, *t* test control vs. primed) and trended toward being higher on ALI day 3 (*p* = 0.15). Expression of EMT-TF slug was detectable on all ALI days in both control and primed cells and decreased over time. In primed cells, compared to control cells, slug expression was significantly higher on ALI days 6 and 10 (∗*p* < 0.05 and ∗∗*p* < 0.01, *t* test control vs. primed). (C) Representative speed maps for unprimed control and TGFβ-primed cells show that the jamming transition is relatively delayed in the primed cells, mimicking the delayed jamming transitions of asthmatic cells. (D) Quantification of average speed in each field of view for unprimed control and primed cells generated from *n* = 3 non-asthmatic donors. As with cells from asthmatic donors, the behavior of the primed cells is variable between donors. (B, D) Blue symbols with an open face denote the unprimed control cells, while blue symbols with a red face denote the primed cells.

On ALI days 6 and 10, compared to the unprimed control cells, the primed cells expressed higher levels of mesenchymal proteins vimentin and slug, mimicking their levels in asthmatic cells. As also demonstrated above (Figures 1 and 2), the levels of these proteins were markedly reduced in unprimed cells as ALI culture progressed, while this reduction was delayed in primed cells (Figures 4A and 4B). Based on the presence of mesenchymal markers in conjunction with the expression of E-cadherin, both unprimed and primed cells appear to be in a hybrid E/M state during early ALI days and undergo MET as ALI culture progresses. In the primed cells, this MET appears to be relatively delayed. The temporal progression of these E/M markers demonstrates that the differences between unprimed and primed cells mimic those between non-asthmatic and asthmatic cells; that is, primed cells recapitulate the delayed MET observed in asthmatic cells. Next, to determine whether this delayed MET in primed cells coincided with a delayed JT, as observed in asthmatic cells (Figure 3), we measured cellular migration on ALI days 3, 6, 10, and 14. The unprimed cells became jammed and non-migratory between days 3 and 6, while the primed cells remained unjammed beyond day 6, continuing even up to day 14 (Figures 4C and 4D). The temporal progression of the JTs from the primed cells recapitulated those seen in asthmatic cells. Together, our data indicate that priming primary basal stem cells with TGF-β1 shifts the phenotype of non-asthmatic cells toward asthma-like cells characterized by delayed MET coupled with delayed JT.

### Priming airway basal stem cells with TGF-β recapitulates asthmatic biophysical phenotype

**Figure 5:**
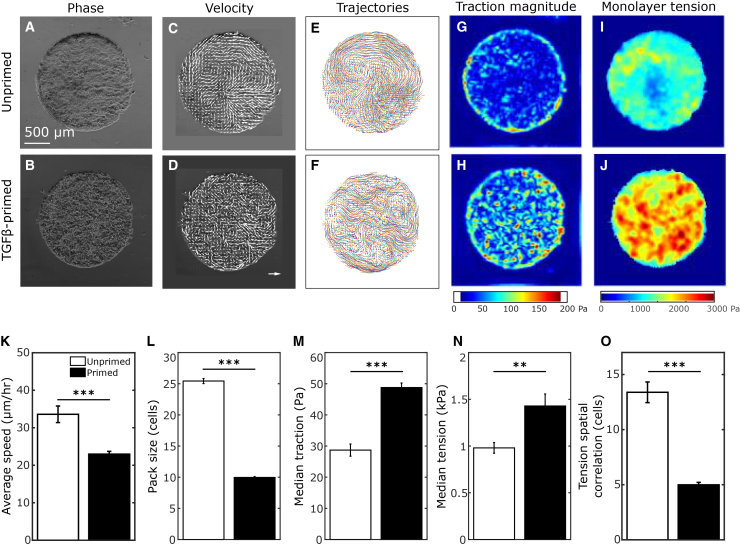
Biophysical characteristics of unprimed versus TGFβ-primed basal stem cells Unprimed control and TGFβ-primed basal stem cells cultured from healthy human donors were micropatterned onto soft polyacrylamide gels and used for traction force microscopy and monolayer stress microscopy to determine cell-substrate and cell-cell forces, respectively. (A and B) Phase contrast microscopy images show snapshots of the micropatterned islands of unprimed control (A) and TGFβ-primed (B) basal stem cells on PA gels. (C and D) Representative velocity vectors (C, D) and integrated trajectories (E, F) for unprimed control and TGFβ-primed monolayers. (G and H) Color maps of tractions exerted by unprimed and TGFβ-primed basal stem cells on their substrates. Color scale is shown at the bottom of (H). (I and O) Color maps of intercellular stresses exerted across cell-cell junctions for unprimed control and TGFβ-primed basal stem cells. Color scale is shown at the bottom of (J). Quantification of average speed (K) and pack size (L, see STAR Methods) shows that unprimed control cells move in faster and larger flocks, while primed cells move in smaller, slower coordinated flocks. Quantification of the median of the traction magnitude (M) and median tension (N) shows increased cell-substrate and intercellular forces exerted by the primed cells. Spatial correlations of tension magnitudes (O) reveal that tension is correlated over a shorter distance in primed cells, indicating that the larger stresses and tractions in these cells were more localized. Quantification of biophysical properties was performed across *n* = 18 unprimed control and *n* = 18 primed islands (K-O; ∗∗*p* < 0.005 and ∗∗∗*p* < 0.0001, *t* test unprimed vs. primed. Data are represented as mean ± SEM).

In addition to a delayed JT, our previous work demonstrated that asthmatic HBE cells exhibited a distinct biophysical phenotype, including elevated monolayer tractions and intercellular stresses.^9^ To determine the extent to which the asthma-like phenotype (Figure 5) in primed cells is accompanied by increased monolayer tractions and intercellular stresses, we measured these in unprimed and primed HBE cells. As performed previously,^73^^,^^83^ HBE cells were seeded on micropatterned collagen islands (1 mm diameter) on soft (4.6 kPa) polyacrylamide gels (Figures 5A and 5B). In this system, only basal cells are present, and cells do not differentiate. To compare the biophysical phenotype between unprimed and primed cells as described above (Figure 4), we tracked cellular movement (Figures 5C–5F) and measured monolayer tractions (Figures 5G and 5H) and stresses (Figures 5I and 5J). In contrast to the elevated speed of cellular migration observed in both asthmatic and primed cells in ALI culture, in this system, we found that, compared to the unprimed cells, the speed of cellular migration was reduced in the primed cells (Figure 5K). We further characterized the cellular movement by measuring the size of coordinated packs of cells, as performed previously.^40^ We detected that the unprimed cells move in larger, faster packs, whereas the primed cells move in smaller, slower packs (Figures 5K and 5L). Primed basal cells exhibited a previously reported asthmatic biophysical signature,^9^ which includes increased traction forces (Figure 5M) and increased intercellular tensions (Figure 5N). Furthermore, primed basal cells exhibited less spatially correlated tensions (Figure 5O), which mirrored their similarly less correlated movement (Figure 5L).

## Discussion

To maintain airway homeostasis and regenerate an intact epithelial layer after injury, airway basal stem cells differentiate into luminal lineages.^46^^,^^84^^,^^85^^,^^86^ Extensive biochemical and molecular studies have been performed to understand cellular processes, particularly in the context of cell type-specific changes during differentiation. However, biophysical characteristics during epithelial cell differentiation, particularly in the context of disease states such as asthma, have only recently begun to be characterized.^9^^,^^41^^,^^87^^,^^88^ Our findings reveal that the differentiation of airway basal stem cells is accompanied by the JT and MET, which occurred simultaneously. In basal stem cells cultured from individuals with asthma, both transitions were markedly delayed, which emphasizes the possibility of delayed transitions as a characteristic of abnormal cellular behavior in asthma pathogenesis.

Our data indicate that as human airway basal stem cells differentiate into a mature epithelium in ALI culture, they display both MET (Figure 1) and JT (Figure 3). The MET process was evidenced by a decrease in mesenchymal markers vimentin and slug and an increase in the epithelial marker e-cadherin, indicating a transition from a hybrid E/M, partially mesenchymal to a fully epithelial state. However, MET was delayed in cells from asthmatic donors, suggesting a disease-specific dysregulation of MET. Moreover, our data reveal that TGF-βR is a key regulator of both MET and JT in HBE cells. The inhibition of TGF-βR activity expedited the delayed MET and JT in asthmatic cells (Figures 2 and 3). Conversely, priming with TGF-β1 delayed both transitions, mimicking the asthmatic phenotype (Figures 4 and 5).

Our investigation into the delayed MET and JT in asthma, both of which are regulated by TGF-βR signaling, aligns with previous findings that TGF-β plays a crucial role in airway basal stem cell dynamics. In particular, TGF-β signaling has been shown to both maintain cellular quiescence during development and also enable regeneration.^89^ Precise control of this pathway is essential because imbalances can lead to basal cell hyperplasia or tumorigenesis, highlighting its significance in airway epithelial homeostasis.^89^^,^^90^ Our findings also underscore the complex role of TGF-βR signaling in airway basal stem cell differentiation. Previous work has shown that the activation of the TGF-βR pathway tightly balances proper epithelial differentiation, particularly through its major canonical pathway, the SMAD pathway, which critically regulates mucociliary differentiation in the airway.^66^ Furthermore, a transcriptomic analysis reveals a downregulation of TGF-β1 and its gene network during mucociliary differentiation in HBE cells within ALI cultures,^45^ while injury repair in alveolar epithelial cells requires initial TGF-βR signaling, followed by its inactivation.^91^ In stem cell systems, TGF-βR inhibition enhances reprogramming of induced pluripotent stem cells (iPSCs)^92^ and promotes the conversion of placental-derived pluripotent stem cells into trophoblast stem cells,^93^ supporting a wide role for TGF-βR signaling in epithelial differentiation.

While the role of TGF-βR is clearly context-dependent,^94^^,^^95^ it is evident that TGF-βR critically orchestrates epithelial-cell differentiation and can trigger EMT, partial EMT, or a hybrid E/M state,^81^^,^^96^^,^^97^ states which confer migratory capabilities on epithelial cells.^21^^,^^74^^,^^80^ Interestingly, HBE cells can become collectively migratory through an unjamming transition, which does not require EMT,^21^ but, according to our RNA seq analysis,^98^^,^^99^ does correspond to an enrichment of the TGF-βR pathway, supporting the regulatory role of TGF-βR in collective phase transitions in HBE cells. In an entirely different system, the mesendoderm of the developing zebrafish embryo, a gradient of Nodal, a highly conserved member of the TGF-β superfamily, has been shown to drive an unjamming transition that is critical for proper patterning.^37^ Together, these studies support a role for TGF-βR signaling in regulating collective migration and phase transitions.

In order to situate the cells along the epithelial-to-mesenchymal spectrum,^4^ we chose to measure the expression of the epithelial marker E-cadherin, the mesenchymal marker vimentin, and the EMT-TF slug. During the differentiation of HBECs in ALI culture, the changes in both E-cadherin and slug were relatively modest, while vimentin expression exhibited the largest changes. Under many experimental conditions vimentin expression was tightly correlated with migration (Figure S2). Vimentin has long been considered a primary marker of mesenchymal cells^100^ and when co-expressed with E-cadherin, indicates that cells are in a hybrid E/M state, which in turn is associated with collective migration.^2^^,^^4^ In a recent report using MCF7 cells, a relatively stationary epithelial cell line, exogenous expression of vimentin initiated and sustained both EMT and migration.^101^ In our studies, in conditions where vimentin expression was high, the HBE cells were unjammed and migratory, consistent with a driving role for vimentin in hybrid E/M collective migration. However, we also observed contradictory instances where the HBE cells were unjammed and migratory in the absence of elevated vimentin levels, suggesting that vimentin is not required for HBE cellular migration. This is consistent with our previous study in which HBE cells became highly unjammed and collectively migratory without the induction of vimentin or other evidence of EMT.^21^

In addition to assessing vimentin to evaluate cell state, we also examined the expression of slug. Slug, encoded by the gene *SNAI2*, is a transcription factor that not only drives EMT but also plays critical roles in cellular migration and survival.^102^ In our study, we find that slug expression decreased over time in ALI but remained detectable even at late ALI days and when cells were jammed and immobile. By contrast, the snail transcription factor, encoded by the gene *SNAI1*, was undetectable in HBE cells even at early ALI days.^21^ Interestingly, the single-cell sequencing-based human lung cell atlas project identified slug, but not snail, as enriched in the airway basal cell population,^103^ while *in vivo* models demonstrate that in the mammary gland, slug is expressed preferentially in the basal cells and plays an important role in their normal stem cell capacity.^104^ These and other studies support a role for slug as a master regulator of differentiation and stem cell fitness.^52^ Slug has also been shown to play an essential role in cutaneous re-epithelialization and wound repair.^105^^,^^106^^,^^107^^,^^108^ The continued expression of slug, even in well-differentiated HBE cells, may therefore contribute to the ability of the airway epithelium to undergo repair and regeneration.

To complement our studies evaluating the effect of TGF-βR inhibition, we generated TGF-β1-primed basal stem cells, which simulated the delayed MET and JT as observed in asthmatic cells (Figures 4 and 5). Beyond the JT, we have previously reported that HBE cells from individuals with asthma generate stronger forces across the epithelial layer, including increased tractions and monolayer stresses^9^ compared to those from donors with no underlying disease. Similar to asthmatic cells, these TGF-β1-primed basal cells exhibited stronger intercellular forces, including increased traction forces, increased intercellular tensions, and less coordinated movement (Figure 5). These data suggest that TGF-β1, which is elevated in the asthmatic airway,^78^ could turn on a cellular memory program in basal stem cells, predisposing them to an asthmatic-like condition characterized by intense cellular forces and uncoordinated movements. This characteristic could make repair of the asthmatic epithelium less efficient when a wound is closed by direct cellular migration. Our results also suggest that TGF-β1-priming may be a strategy to allow investigations into behaviors of asthmatic-like cells using cells derived from healthy individuals. This may benefit pilot studies or experiments where cost scales with the number of precious human samples. Our study, along with previous work, demonstrates that compared to non-diseased donors, donor-to-donor heterogeneity is greater in asthmatic cells,^99^ supporting the value of the priming strategy in minimizing variability.

Our study is not without limitations. The use of *in vitro* culture systems and primary cells from a limited number of asthmatic donors calls for further validation in more physiologically representative models, including *in vivo* studies, to enhance the physiological relevance of our findings. Such *in vivo* studies are essential to validate the significance of our findings within the complex environment of the human airway, which is influenced by numerous factors and signaling pathways, such as the interplay of the Wnt pathway with TGF-βR signaling in regulating cellular transitions.^109^ Interestingly, recent advances have allowed measurement of collective cellular migration in explanted murine airways following injury,^110^^,^^111^ demonstrating the feasibility of evaluating JTs in intact tissues. These future directions will be instrumental for unraveling the molecular mechanisms of JT and their implications in airway diseases like asthma. Our findings regarding the role of TGF-βR signaling in the differentiation of HBE cells and its regulatory role in the delayed MET and JT in asthma complement the recent advances in utilizing iPSC-derived airway basal cells (iBCs). The use of iBCs presents a promising avenue for understanding airway diseases, including asthma.^112^ Specifically, our work underscores the therapeutic potential of targeting TGF-βR signaling pathways, a key pathway in the maintenance and expansion of iBCs.^113^ This suggests that our *in vitro* findings in HBE cells in ALI culture could be translated into iBC systems, potentially creating a more disease-relevant platform for studying the pathophysiology of airway diseases. The next steps could focus on integrating our insights on signaling pathways with iBC-derived cells in ALI cultures, to enhance our understanding of precise pathologic mechanisms underlying chronic airway diseases.

The differentiation of airway basal stem cells into mature epithelial cells involves coordinated changes in cell state, dynamics, and molecular mechanisms. In this study, we show that as cells differentiate, they undergo a transition from an unjammed, migratory state to a jammed, solid-like state. This transition coincides temporally with features of the MET. However, this temporal alignment does not necessarily imply that the JT and MET are mechanistically identical. For example, our previous work demonstrated that UJT can occur independently of EMT, indicating that these are distinct.^21^ Together, our work highlights that epithelial plasticity involves complex and context-dependent processes that may converge or diverge under specific physiological conditions, without being mechanistically identical. Furthermore, our data point toward the hypothesis that jamming and unjamming transitions are not necessarily simply inverse processes.

In conclusion, our study unveils the cellular plasticity and dynamics that govern the differentiation of human airway basal stem cells into luminal lineages through the JT and EMT, both precisely regulated by the activation of TGF-βR. We demonstrate that TGF-βR is central to regulating both epithelial-mesenchymal and JTs crucial for epithelial plasticity, offering mechanistic insights into the fate of airway epithelial cells, which is central in asthma pathophysiology and airway epithelial biology.

### Limitations of the study

Our study identifies the concurrence of the jamming and the METs and shows that TGF-βR signaling is involved in both transitions during differentiation. However, it remains unknown whether jamming and MET are interdependent or simply concurrent downstream of TGF-βR signaling. In order to define this mechanistic link, future studies will directly control either cell jamming or the pathways that directly regulate MET. Furthermore, we show that the inhibition of TGF-βR signaling can rescue the delayed jamming and partial MET phenotype of HBE cells derived from patients with asthma. However, the underlying signaling mechanism is unknown. Future studies will examine both canonical and non-canonical TGF-βR signaling.

## Resource availability

### Lead contact

Further information and requests for resources and reagents should be directed to Jin-Ah Park (jpark@hsph.harvard.edu).

### Materials availability

This study did not generate new unique reagents.

### Data and code availability

• The RNA-seq data generated (raw and processed files) for this manuscript are available in the ArrayExpress maintained by the European Bioinformatics Institute (EMBL-EBI) under accession number (EMBL-EBI: E-MTAB-16209). The accession number is listed in the key resources table.
• Data, both raw and analyzed, that comprise the graphs within this manuscript and other findings of this study are available from the corresponding author upon request. The source data underlying all graphs, along with uncropped western blots, are provided as a Source Data file. The code used to process the data and generate the graphs within this manuscript is available from the corresponding author upon request.
• Any additional information required to reanalyze the data reported in this paper is available from the lead contact upon request.

## Acknowledgments

This study was funded by 10.13039/100000050NHLBI: R01HL148152 (J-AP), 5P01HL152953(J-AP), T32HL007118 (JAM and TKP), 10.13039/100000066NIEHS: P30ES000002 (J-AP), 10.13039/501100002424FujiFilm Corporation (CM), 10.13039/100005834Francis Family Foundation (JAM), and 10.13039/100005588Wesleyan University (JAM).

## Author contributions

Conceptualization: J.A.M. and J-A.P.; data curation: J.A.M., J.N., S.K., and J.D.C.; formal analysis: J.A.M., J.N., and S.K.; investigation: J.A.M., J.N., R.R.B., M.A.N., S.K., C.M., and M.J.O.; methodology: J.A.M. and J-A.P.; resources: J.A.M., J.D.C., and J-A.P.; software: J.A.M. and J.N.; supervision: J.A.M. and J-A.P.; validation: J.A.M.; visualization: J.A.M., J.N., R.R.B., and S.K.; writing – original draft: J.A.M. and J-A.P.; writing – review and editing: J.A.M., J.N., and J-A.P.

## Declaration of interests

The authors declare no competing interests.

## STAR★Methods

### Key resources table

**Table:** 

REAGENT or RESOURCE	SOURCE	IDENTIFIER
**Antibodies**		

Rabbit monoclonal anti-E-cadherin (1:10,000)	Cell Signaling Technology	Cat #3195; RRID:AB_2291471
Rabbit monoclonal anti-slug (1:1000)	Cell Signaling Technology	Cat #9585; RRID:AB_2239535
Rabbit monoclonal anti-vimentin (1:1000)	Cell Signaling Technology	Cat #5741; RRID:AB_10695459
Rabbit monoclonal anti-GAPDH (1:5000)	Cell Signaling Technology	Cat #5174; RRID:AB_10622025
HRP-linked goat anti-rabbit IgG (1:2500)	Cell Signaling Technology	Cat # 7074; RRID:AB_2099233
Rabbit monoclonal anti-p63 (1:400)	Cell Signaling Technology	Cat #13109; RRID:AB_2637091
Mouse monoclonal anti-MUC5AC (45M1) (1:100)	ThermoFisher Scientific	Cat# MA5-12178; RRID:AB_10978001
Mouse monoclonal anti-beta IV tubulin (1:400)	Sigma	Cat #T7941; RRID:AB_261775
Alexa Fluor 488 conjugated goat anti-rabbit	Invitrogen	Cat# A32731
Alexa Fluor 488 conjugated goat anti-mouse	Invitrogen	Cat# A32742

**Chemicals, peptides, and recombinant proteins**		

Paraformaldehyde, 16%	Electron Microscopy Sciences	Cat# 15710
Phalloidin-594 (1:400)	Invitrogen	Cat #A12381
Hoechst 33342 (1:5000)	ThermoFisher Scientific	Cat # 62249
TGF-β receptor inhibitor SB-431542	Tocris	Cat # 1614; CAS # 301836-41-9
Acrylamide	BioRAD	Cat # 161-0140
Bisacrylamide	BioRAD	Cat # 161-0142
TEMED	BioRAD	Cat # 1610800
sulfo-SANPAH	Proteochem	Cat #c1111-100mg; CAS Number: 102568-43-4
Bovine collagen I (PureCol)	Advanced Biomatrix	Cat # #5005
Fluorescent beads	ThermoFisher Scientific	Cat #F8812
Nystatin	Sigma	Cat # *N*-1638
Retinoic acid	Sigma	Cat # R-2625
DTT (Dithiothreitol)	Sigma	Cat #D9779
2X Laemmli sample buffer	Biorad	Cat # 1610737

**Critical commercial assays**		

TRIzol Plus RNA Purification Kit	Invitrogen	Cat # 12183555

**Deposited data**		

RNA sequencing data at ALI days 0, 1, 7, 14	This paper	ArrayExpress Accession # E-MTAB-16209

**Experimental models: Cell lines**		

Primary human bronchial epithelial cells	Marsico Lung Institute/Cystic Fibrosis Research Center, University of North Carolina	N/A

**Software and algorithms**		

CellPose	Stringer et al.^114^	https://www.cellpose.org/
ImageJ	Schindelin et al.^115^	https://imagej.net/ij/
MorphoLibJ Plugin for ImageJ	Legland et al.^116^	https://imagej.net/plugins/morpholibj
LabelstoROI Plugin for ImageJ	Waisman et al.^117^	https://labelstorois.github.io/
Galaxy platform	Afgan et al.^118^	https://galaxyproject.org/
FastQC	Babraham Bioinformatics	https://www.bioinformatics.babraham.ac.uk/projects/fastqc/
Bowtie2	Langmead et al.^119^	http://bowtie-bio.sourceforge.net/bowtie2/index.shtml
Samtools	Li et al.^120^	http://samtools.sourceforge.net/
FeatureCounts	Liao et al.^121^	https://rnnh.github.io/bioinfo-notebook/docs/featureCounts.html
Ensembl genome browser	Dyer et al.^122^	https://useast.ensembl.org/index.html

**Other**		

DMEM (high glucose)	Corning	Cat #10-013-CV
BEGM Bullet Kit (media & supplements)	Lonza	Cat # CC-3170
SDS-PAGE mini-protean protein gels	Biorad	Cat # 4561036
SDS-PAGE criterion protein gels	Biorad	Cat # 5671035

### Experimental model and study participant details

Passage 2 primary human bronchial epithelial (HBE) cells were differentiated in air-liquid interface (ALI) culture, as detailed below and described previously.^9^^,^^21^^,^^32^^,^^36^^,^^43^^,^^47^^,^^123^^,^^124^^,^^125^^,^^126^ Initially, primary HBE cells were isolated at Passage 0 at the Marsico Lung Institute/Cystic Fibrosis Research Center, University of North Carolina, Chapel Hill. These cells were obtained from human lungs deemed unsuitable for transplantation under protocol #03–1396, approved by the University of North Carolina at Chapel Hill Biomedical Institutional Review Board. Donors were either categorized as “non-asthmatic,” which were non-smokers without a history of chronic lung disease, or as “asthmatic,” which were patients previously diagnosed with asthma. Donor demographic information is available upon request. The cells were obtained at Passage 0 or Passage 1 and expanded to Passage 2 in our laboratory and utilized for all experiments.

Passage 2 primary HBE cells were seeded onto type I collagen-coated (0.05 mg/mL) transwell inserts (Corning, 12 mm, 0.4 μm pore, polyester) and grown in submerged conditions for 4–6 days. Cells were cultured in a 1:1 mix of DMEM (high glucose, 4.5 g/L) and bronchial epithelial basal medium (BEBM, Lonza), supplemented with bovine pituitary extract (BPE, 52 μg/mL), epidermal growth factor (EGF, 0.5 ng/mL), epinephrine (0.5 μg/mL), hydrocortisone (0.5 μg/mL), insulin (5 μg/mL), triiodothyronine (6.5 ng/mL), transferrin (10 μg/mL), gentamicin (50 μg/mL), amphotericin-B (50 ng/mL), bovine serum albumin (1.5 μg/mL), nystatin (20 units/ml), and retinoic acid (50 nM). Throughout the culture period, HBE cells were maintained in this defined, serum-free media. Once a confluent layer was achieved, the medium was removed from the apical surface to initiate the ALI condition. While differentiation into a fully pseudostratified epithelium that mimics the cellular architecture and composition of the intact human airway requires at least 14 days in culture, in this study cells were primarily used at earlier days 3, 6, and 10 to study phase transitions during early differentiation. Prior to timelapse imaging experiments and subsequent collection of cells for detection of proteins, the cells were maintained overnight in medium depleted of EGF, BPE, and hydrocortisone. Experiments were conducted with primary bronchial epithelial cells from at least *n* = 3 non-asthmatic donors and from *n* = 3 or *n* = 6 asthmatic donors in independent experiments.

### Method details

#### Immunocytochemistry

HBE differentiation was confirmed through immunofluorescent labeling of differentiation markers. As described previously,^47^ HBE cells were fixed in 4% paraformaldehyde (Electron Microscopy Sciences) for 10 min and washed 3x in PBS. Sections of transwells were removed from their plastic housing for staining. These sections were incubated in 0.2% Triton X-100 to permeabilize for 10 min, followed by blocking (10% normal goat serum, 1% BSA) for 1 h. Sections were incubated with primary antibodies overnight at 4°C, followed by 3x washes in PBS and incubation with secondary antibodies (Alexa Fluor 488 conjugated goat anti-rabbit or anti-mouse, Invitrogen). Sections were counterstained for F-actin (Alexa Fluor 594 phalloidin, 1:400) and DNA (Hoechst, 1:5000) and mounted with Vectashield mounting medium. Fluorescent z-stacks were acquired using Zen Blue 2.0 software on a Zeiss Axio Observer Z1 with an Apoptome 2 module. Side view images were reconstructed in ImageJ.

#### RNA-sequencing analysis

Total RNA was isolated from HBE cells frozen on ALI days 0, 1, 7, and 14 using the TRIzol Plus RNA Purification Kit (Invitrogen) according to the manufacturer’s instructions. Quality of RNA was assessed using the Nanodrop 8000 spectrophotometer. Sequencing libraries were constructed with a Watchmaker Genomics kit. RNA sequencing was performed on a NovaSeq PE150. Library construction and sequencing was performed at the Oklahoma Medical Research Foundation Clinical Genomics Center.

Downstream bioinformatics analysis of RNA sequencing data was performed using the Galaxy platform.^118^ Quality check of raw sequencing reads was conducted with the FastQC tool (https://www.bioinformatics.babraham.ac.uk/projects/fastqc/). To address high adaptor content, 91 base pairs were trimmed from the 3′ end of each read. Reads were then aligned to the human reference genome (Homo_sapiens.GRCh38.dna.toplevel.fa.gz) using Bowtie2 with default parameters.^119^ Mapping statistics were evaluated using the Samtools flagstat program.^120^ Gene-level quantification was performed using featureCounts with default parameters,^121^^,^^127^ guided by the corresponding GTF annotation file (Homo_sapiens.GRCh38.113.gtf.gz). Both the reference genome and the GTF file were obtained from the Ensembl genome browser^122^ (https://useast.ensembl.org/index.html). Raw count files from individual samples were merged into a single count matrix, which was then used to construct a DESeq2 object in R for differential expression analysis, using day as the design variable.^128^ Normalized expression values generated by DESeq2 were used to visualize gene expression dynamics over time. Individual replicates were plotted as colored points, while the mean expression at each time point is shown as a black dot connected by lines across different time points using the ggplot() function in R.^129^ Standard error of the mean was calculated at each time point to generate error bars.

#### Inhibitor treatment

HBE cells were treated with TGF-β receptor inhibitor SB-431542 (Tocris) for 3 days prior to imaging and collection. SB-431542 (abbreviated as SB) is a selective and potent inhibitor of TGF-β receptor I (TGF-βRI) ALK5 along with ALK4 and ALK7.^130^ SB was reconstituted to 10 mM in DMSO and used at 5 μM. Vehicle control cells were treated with an identical volume of DMSO as SB.

#### Generation of primed HBE cells

A single vial of passage 1 HBE cells was split into multiple flasks which were grown in parallel for 3–5 days until ∼50% confluence was reached. At this point, flasks were either incubated with 0.3 ng/mL TGF-β1 or vehicle and cultured for an additional 2–3 days until ∼90% confluence was reached, at which point cells were passaged and frozen at passage 2 using standard practices.^43^ We generated primed and unprimed vehicle control cells from *n* = 3 non-asthmatic donors.

#### Protein expression analysis

We detected protein levels by western blot analysis as described previously.^124^ Cell lysates were collected into 150 μL 2x Laemmli buffer with 1M DTT. The following antibodies and dilutions were used: primary antibodies were diluted in 5% skim milk or 5% BSA according to the manufacturer’s instructions: E-cadherin (1:10,000), slug (1:1000), vimentin (1:1000), GAPDH (1:5000); secondary antibody was diluted in 3% skim milk: anti-rabbit IgG, HRP-linked (1:2500); all from Cell Signaling Technology. We report fold-changes of normalized protein levels compared to the average expression of that protein on ALI day 3 for all non-asthmatic donors. Uncropped blots and corresponding white light images to show the visible ladder are provided as a supplemental data file (see Data S1/Methods S1).

#### Live imaging and analysis of dynamics

To determine cellular dynamics of HBE cells in ALI culture, time-lapse movies were acquired and analyzed as described previously.^21^ Phase contrast images were acquired using Zen Blue 2.0 software on a Zeiss Axio Observer Z1 with stage incubator (37°C, 5% CO_2_). Images were taken every 6 min for 4 h. Custom software using an optical flow algorithm written in MATLAB (R2019a) was used to analyze the time-lapse movies. Flow fields were calculated using MATLAB’s OpticalFlowFarneback function. From these flow fields, trajectories were obtained by seeding the first image in the sequence using a square grid with spacing comparable to the cell size and then forward-integrating the flow fields. Average speed was then calculated from the displacement of individual trajectories during the time-lapse.

#### Cell shape analysis

Cell shape data was obtained from segmentation of phase contrast images using the open-source software CellPose.^114^^,^^131^ CellPose machine learning models were trained on a subset of images with manually annotated cell outlines. These models were used for bulk processing of images using a custom python script. Segmentation was exported as the native NPY CellPose file and as a PNG image of the masks. Cell shape data was obtained from the PNG files in FIJI (ImageJ)^115^ as described previously.^132^ Connected components labeling in the MorphoLibJ plugin^116^ identified the cell outlines from CellPose output. Labels of cells were converted to regions of interest (ROIs) and measured using the LabelstoROI plugin.^117^

#### Traction force and monolayer stress microscopy

Polyacrylamide substrates of 4.8 kPa Young’s modulus and 100 μm thickness were prepared by mixing a solution containing 7.5% weight/volume (w/v) acrylamide, 0.054% w/v bisacrylamide, 5.5 w/v ammonium persulfate, 0.05% TEMED (all from BioRad), and 0.014% w/v 0.5 μm fluorescent particles (ThermoFisher Scientific), pipetting the solution onto 35 mm diameter glass-bottom dishes, and centrifuging upside down during polymerization to locate fluorescent particles to the top of each gel, as described previously.^83^ 1 mm islands of collagen I were micropatterned onto the polyacrylamide substrates as described previously.^83^ Briefly, silicone masks with 1 mm holes were placed on top of the polyacrylamide substrates and sulfo-SANPAH (Proteochem) was to covalently attach collagen I. 1 mL of 0.1 mg/mL solution of collagen I (Advanced Biomatrix) was added to each dish and kept at 4°C overnight. The collagen was rinsed with PBS, cells were seeded on the substrates, and the masks were removed, leaving a confluent island of cells. One day later, the cells and fluorescent particles were imaged in phase contrast and fluorescent modes using a Leica DMI6000b fluorescent microscope with a 5× objective and Hamamatsu Orca Flash 4.0 camera. Following imaging, the cells were released from the substrate with trypsin, and a set of images were acquired that served as the undeformed reference state for traction force microscopy.

Cell velocities were calculated by applying digital image correlation to the phase contrast images, and trajectories were approximated from the velocity data by interpolation over time, as described previously.^133^ To compute tractions and stresses, the images of fluorescent particles were analyzed by image correlation as described previously.^133^ Tractions were then calculated by unconstrained Fourier transform traction cytometry accounting for the finite substrate thickness.^134^^,^^135^^,^^136^ Next, monolayer stress microscopy^133^^,^^137^^,^^138^ was used to calculate the intercellular stress. The stress is a tensor in the plane of the cell monolayer; following prior studies, we computed the average principal stress, which we refer to as the monolayer tension.^138^ A spatial autocorrelation of the monolayer tension was computed as described previously,^139^ and the correlation length was defined as the distance at which the normalized spatial correlation decreased by 50%.

### Quantification and statistical analyses

All experiments in ALI cultured were repeated independently with HBE cells derived from at least three donors with two biological replicates per condition and timepoint. The number of donors for each experiment is indicated in the relevant Figure caption. The experiments on PA gels used one donor with unprimed control and primed cells, and were repeated across two independent experiments with measurements from n = 8–10 islands per experimental condition.

Protein measurements were repeated in *n* = 3 or *n* = 6 donors from independent experiments with two biological replicates per condition and timepoint. Western blots were loaded in parallel, with gel running and imaging carried out under with identical conditions. Each blot was normalized to its own internal loading control of GAPDH. Expression of each protein across donors and days was then normalized to the average value of that protein in non-asthmatic cells on ALI day 3. These normalized protein levels were then compared between non-asthmatic and asthmatic (Figure 1), between vehicle- and SB-treated (Figure 2), or between unprimed and primed (Figure 4), using a two-tailed *t* test for the data at each ALI day. The blots show representative data which was consistent across the donors used.

To determine the effect of SB treatment on cellular motility and shape, average speed and average aspect ratio were determined for each timelapse movie, as described above. The relationship between the average speed and aspect ratio of was determined using Pearson’s linear correlation coefficient. Each vehicle-treated donor on each day was then determined to be considered “migratory” if the average speed was over 1.8 μm/h. For these conditions, speed was then compared between vehicle-treated and SB-treated cells using a paired *t* test.

Biophysical characteristics of unprimed control and primed HBE cells on PA gels were assessed as described above, and were compared using two-tailed t-tests.

## Supplemental information

Document S1. Figures S1–S2 and Data S1/Methods S1
