# Supplementary material for: Concurrent jamming and mesenchymal-to-epithelial transitions during airway basal stem cell differentiation
Source: iScience. 2026 Apr 28;29(6):115935. doi: 10.1016/j.isci.2026.115935 (PMC13213797; doi:10.1016/j.isci.2026.115935)

iScience, Volume *29*

Supplemental information

Concurrent jamming and mesenchymal-to-epithelial transitions during airway basal stem cell differentiation

Jennifer A. Mitchel, Jacob Notbohm, Reverie R. Brown, Mikoto A. Nakamura, Siddhant Kalra, Joseph D. Coolon, Chimwemwe Mwase, Michael J. O'Sullivan, and Jin-Ah Park

Supplemental Figures

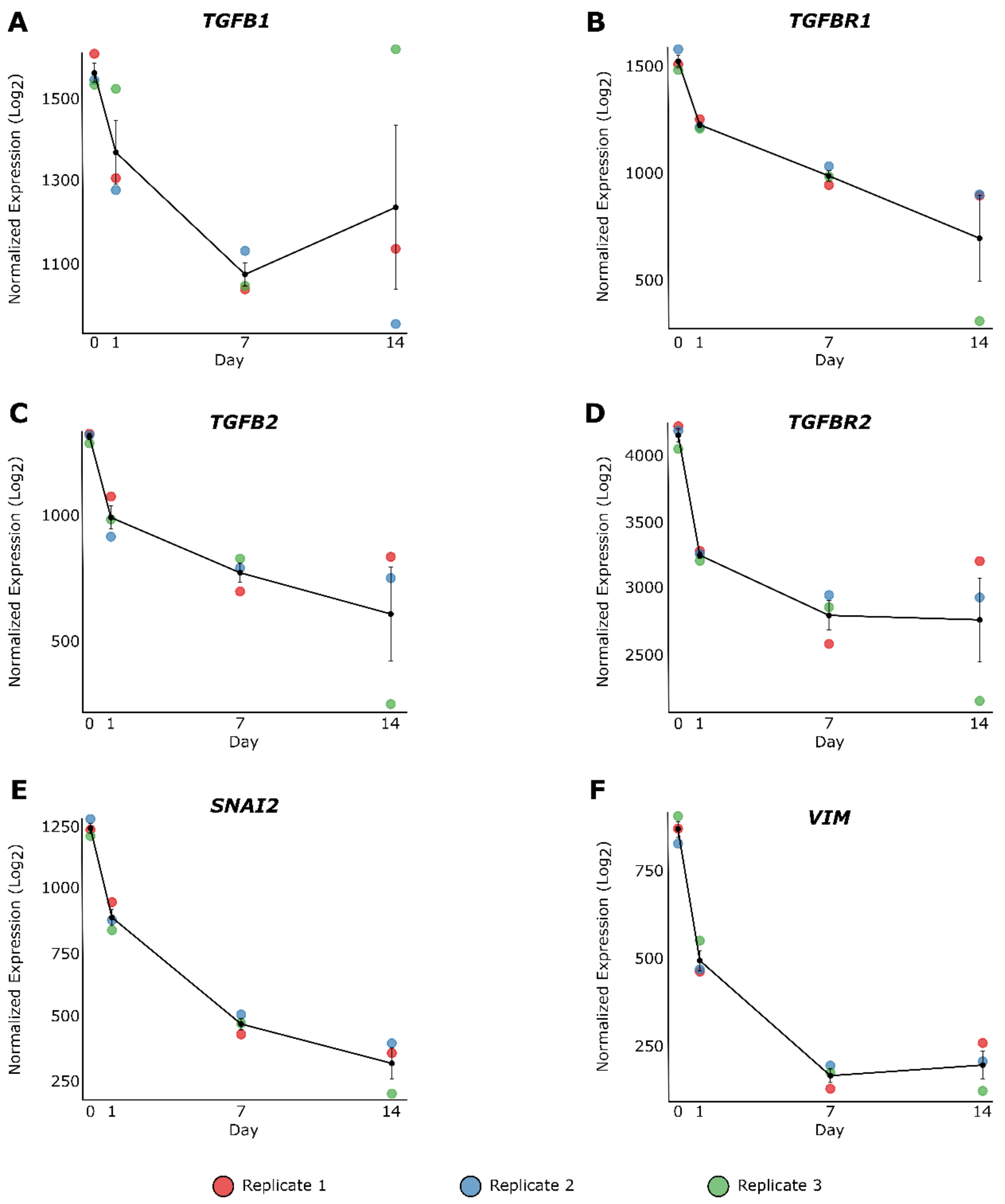

Fig. S1. Expression of genes implicated for TGFβR activation and mesenchymal state decreases over differentiation of HBE cells. HBE cells were collected on ALI days 0, 1, 7, and 14 for RNA-seq analysis. Normalized gene expression for genes of interest are shown over ALI day. Expression of *TGFB1*, *TGFBR1*, *TGFB2*, *TGFBR2, SNAI2,* and *VIM* decreases over ALI day during differentiation.

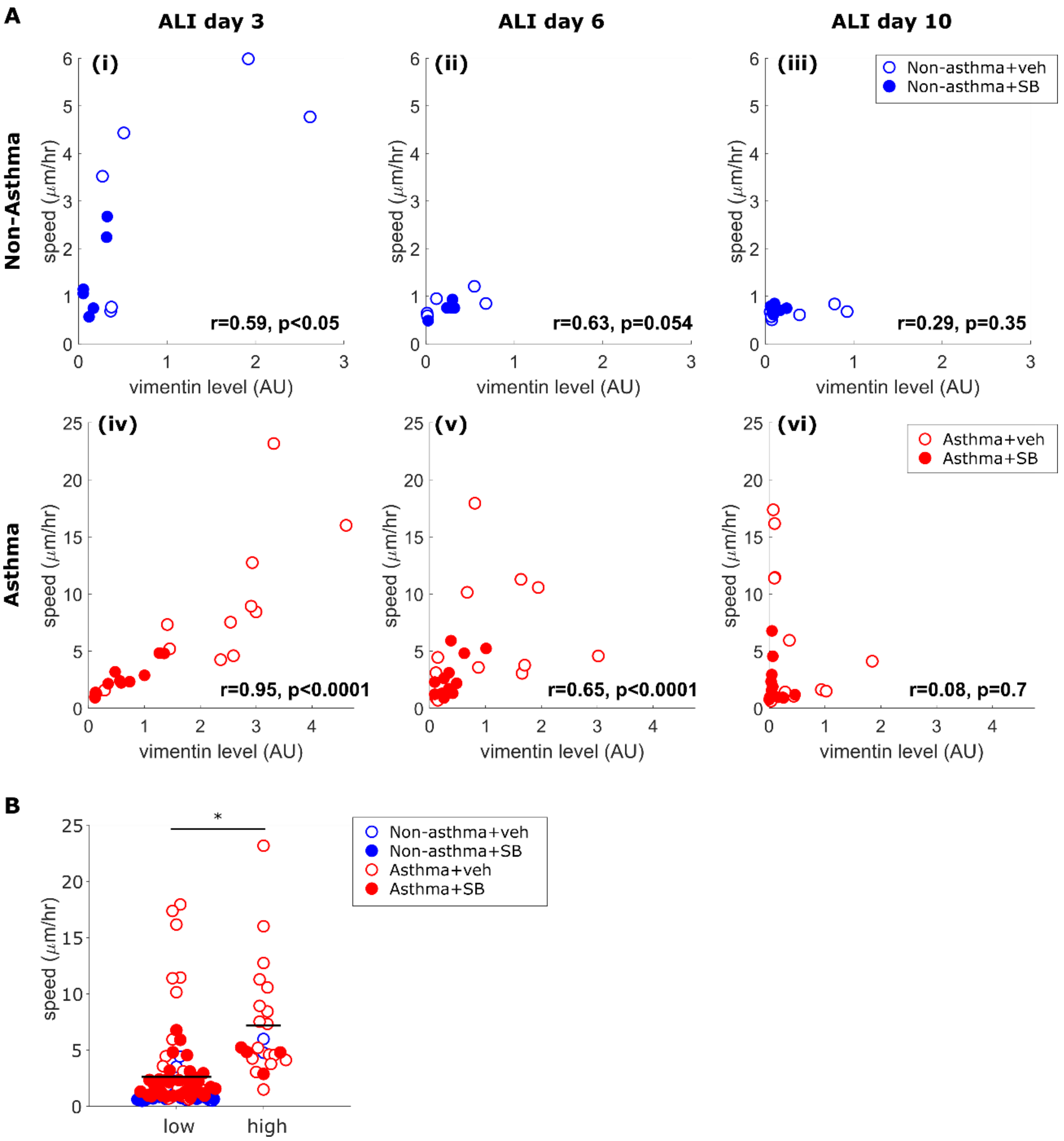

Fig. S2. Vimentin expression is not necessary but may be sufficient for epithelial unjamming. For each well, overall vimentin expression and average speed were calculated. (A) For each of 2 biological replicates for n=3 non-asthmatic and n=6 asthmatic donors, vimentin levels (normalized the average expression at ALI day 3 across all vehicle-treated non-asthmatic donors) are plotted versus the corresponding average speed. For non-asthmatic cells on ALI day 3 (panel i) and asthmatic cells on ALI days 3 and 6 (panels iv and v), baseline vimentin expression (open symbols) is relatively high and there is a significant correlation between vimentin level and average speed (reported in each panel is the correlation coefficient, r, and the p-value calculated from Spearman's correlation). However, for non-asthmatic cells on ALI days 6 and 10 (panels ii and iii), and asthmatic cells on ALI day 10 (panel vi), baseline expression was relatively low, and vimentin level and speed are no longer correlated. Interestingly, 3 asthmatic donors display relatively high speeds but undetectable or low vimentin

expression. (B) Samples were divided in “low” and “high” categories by the amount of vimentin expressed, such that “low” samples correspond to less vimentin than the average expression on ALI day 3 for vehicle-treated non-asthmatic donors, and “high” samples correspond to greater vimentin expression. Across all conditions, the low vimentin-expressing conditions exhibited significantly lower speeds ($p<0.0001$, two-tailed t-test).

Data S1/Methods S1: Uncropped Western blots and corresponding white light images

Figure 1 – E-cadherin (135 kDa)

Top: uncropped western blot with labels placed according to visible light image

Bottom: uncropped visible light image with labeled protein standards

Ladder used: Biorad Precision Plus Protein Standard, Dual color (1610374)

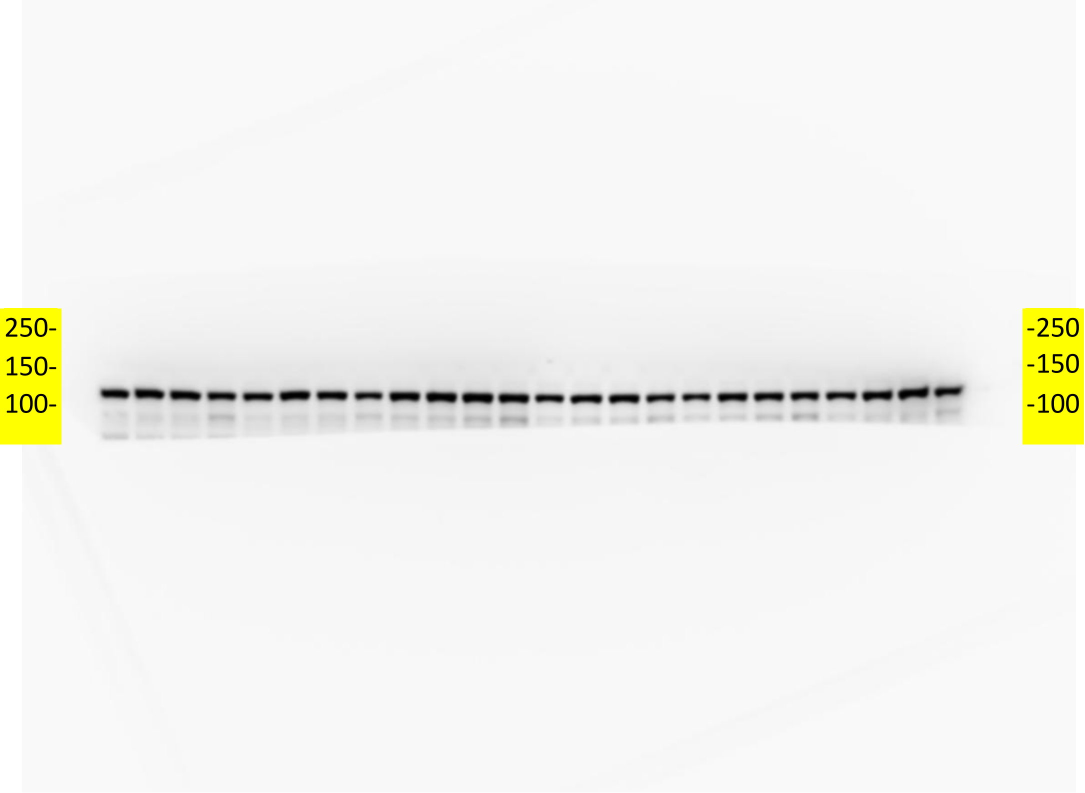

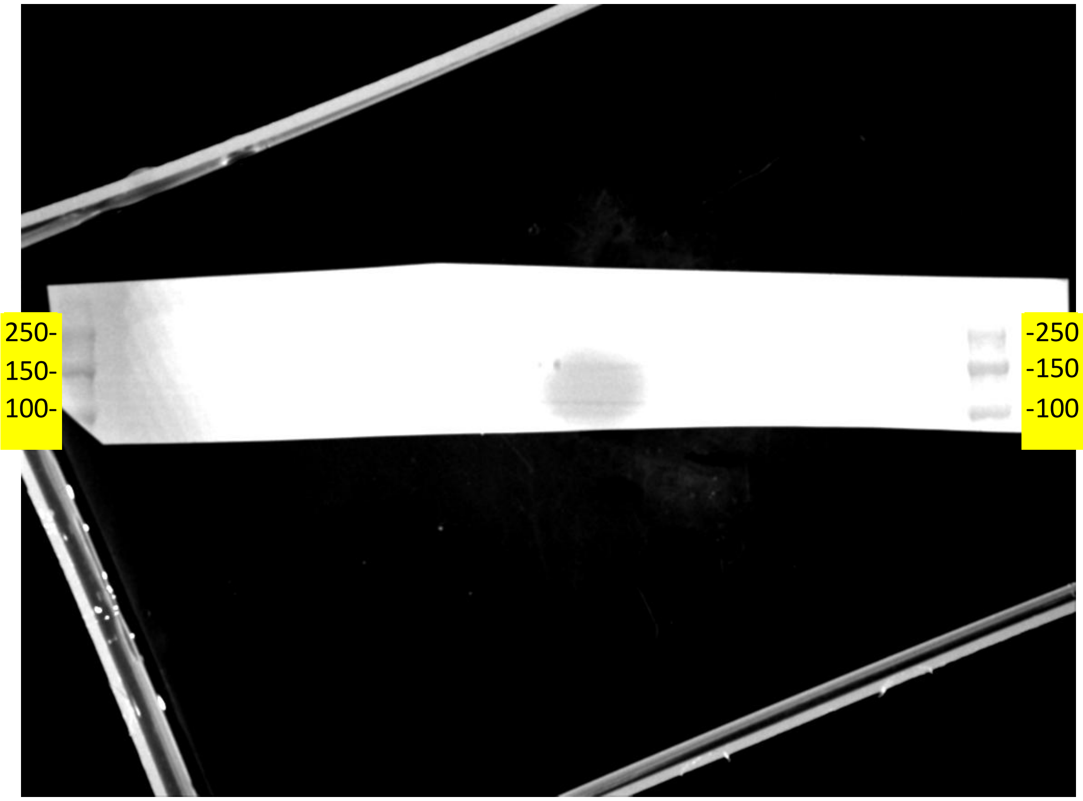

Figure 1 – Vimentin (60 kDa)

Top: uncropped western blot with labels placed according to visible light image

Bottom: uncropped visible light image with labeled protein standards

Ladder used: Biorad Precision Plus Protein Standard, Dual color (1610374)

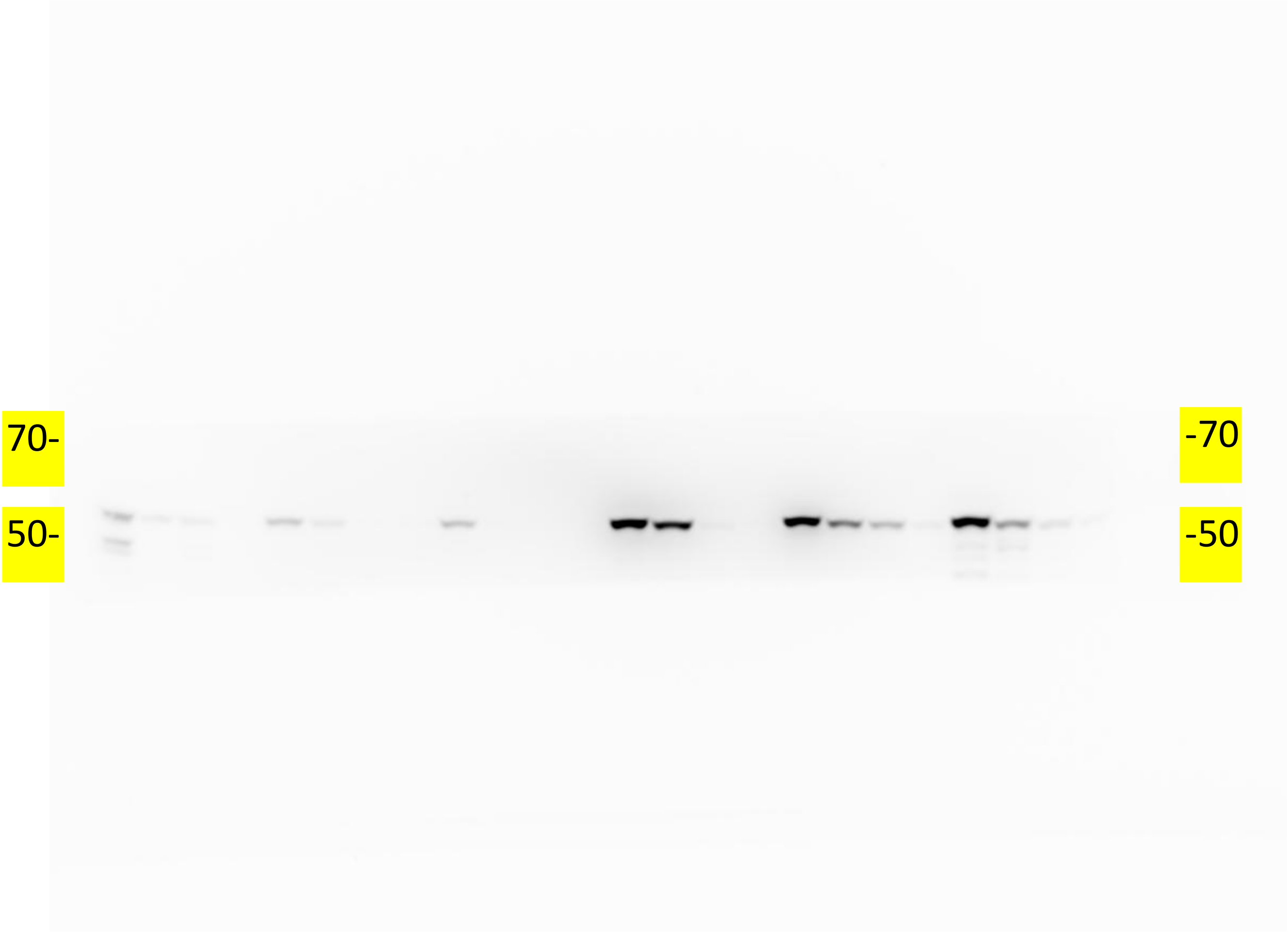

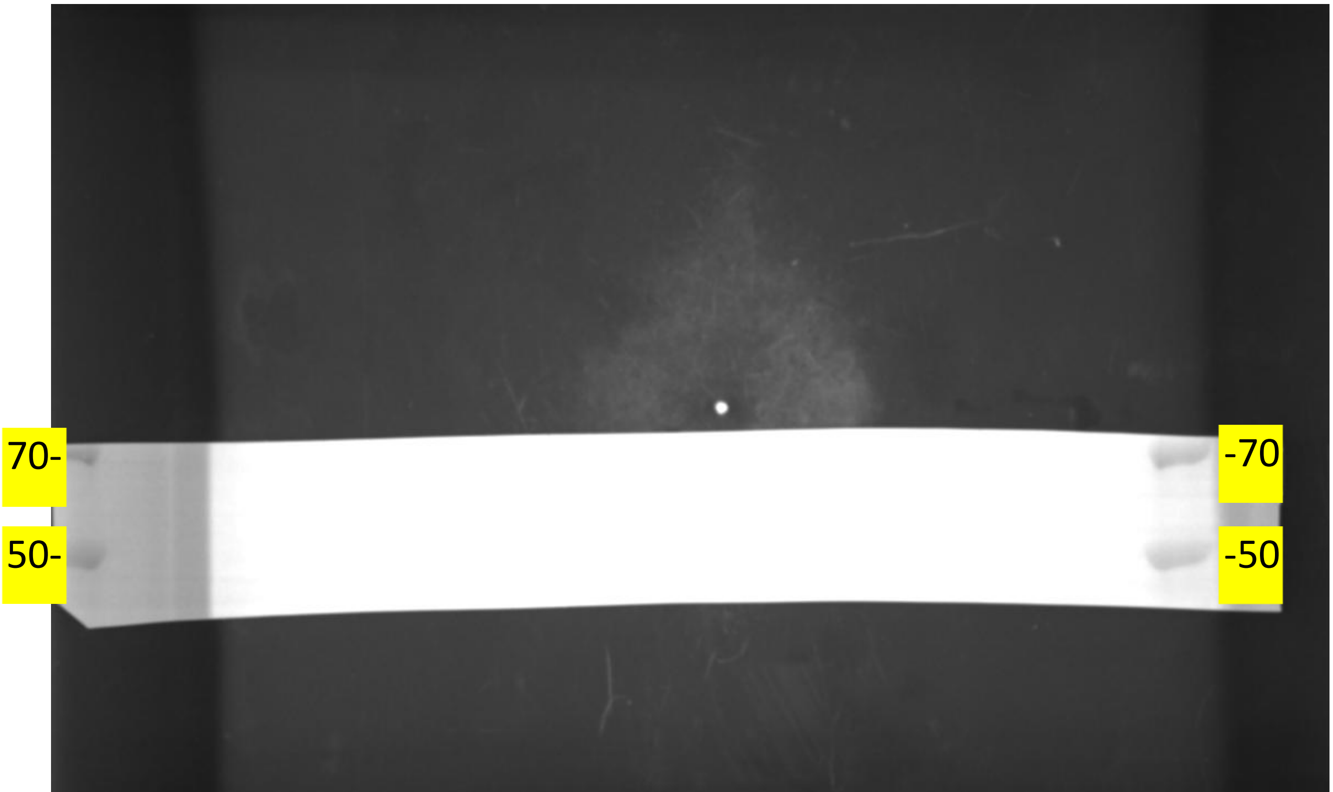

Figure 1 – Slug (30 kDa)
Top: uncropped western blot with labels placed according to visible light image
Bottom: uncropped visible light image with labeled protein standards
Ladder used: Biorad Precision Plus Protein Standard, Dual color (1610374)

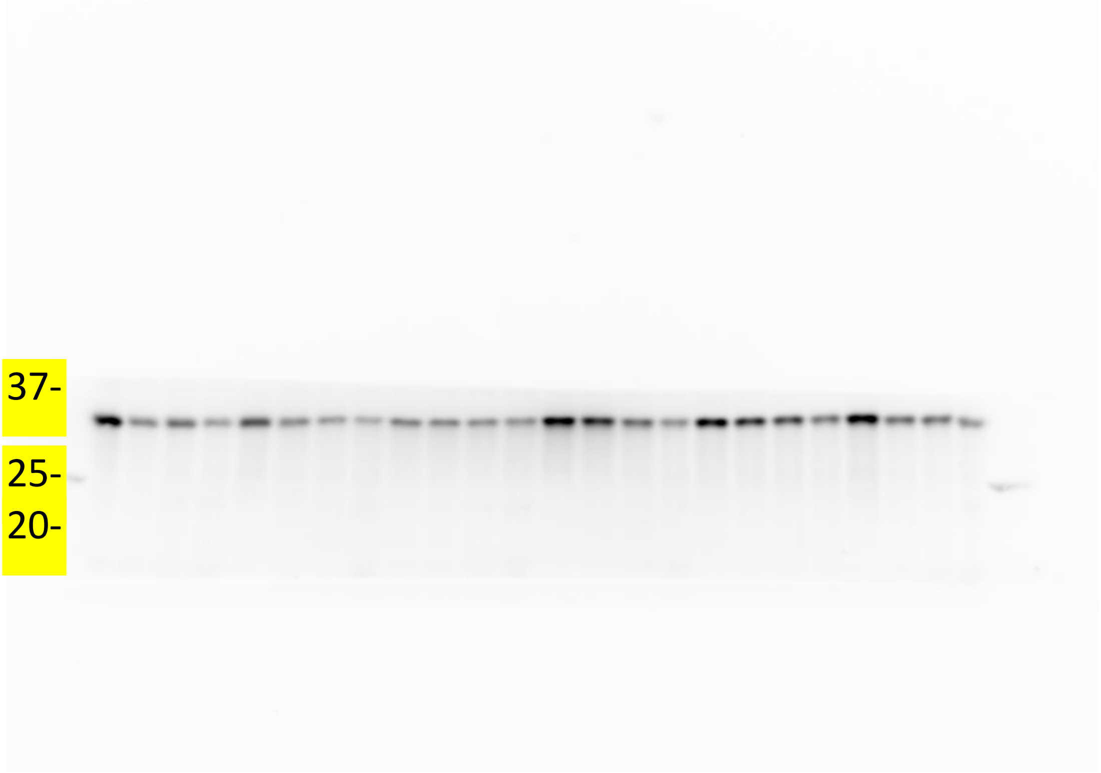

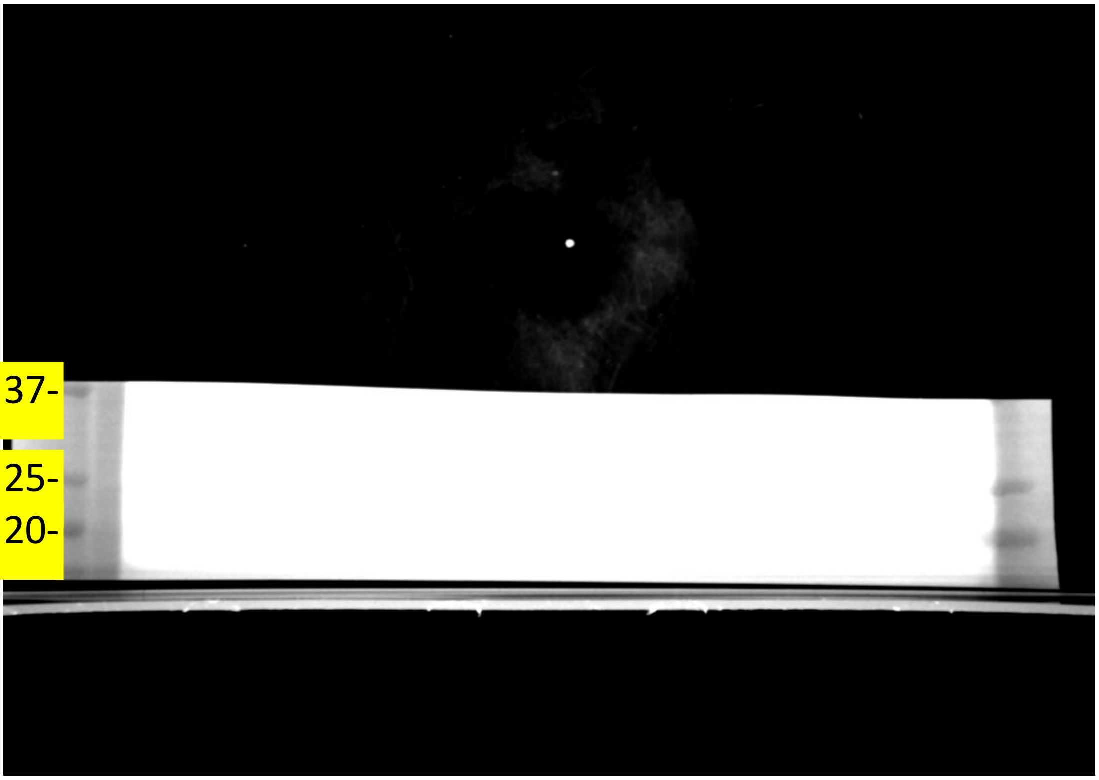

Figure 1 – GAPDH (34 kDa)
Top: uncropped western blot with labels placed according to visible light image
Bottom: uncropped visible light image with labeled protein standards
Ladder used: Biorad Precision Plus Protein Standard, Dual color (1610374)

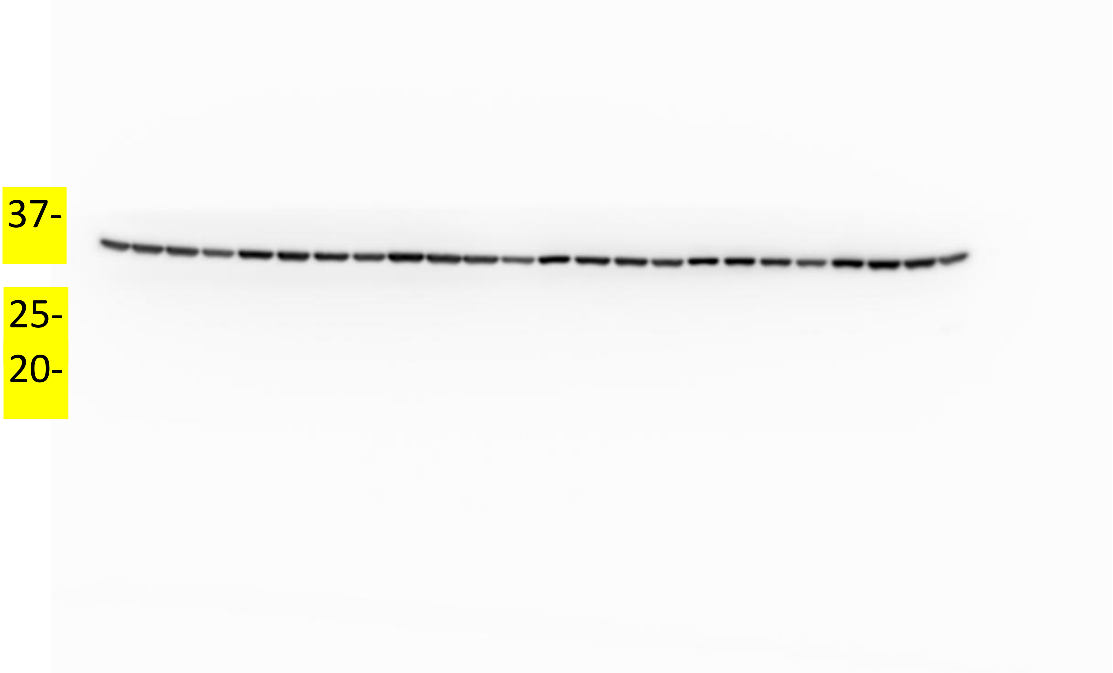

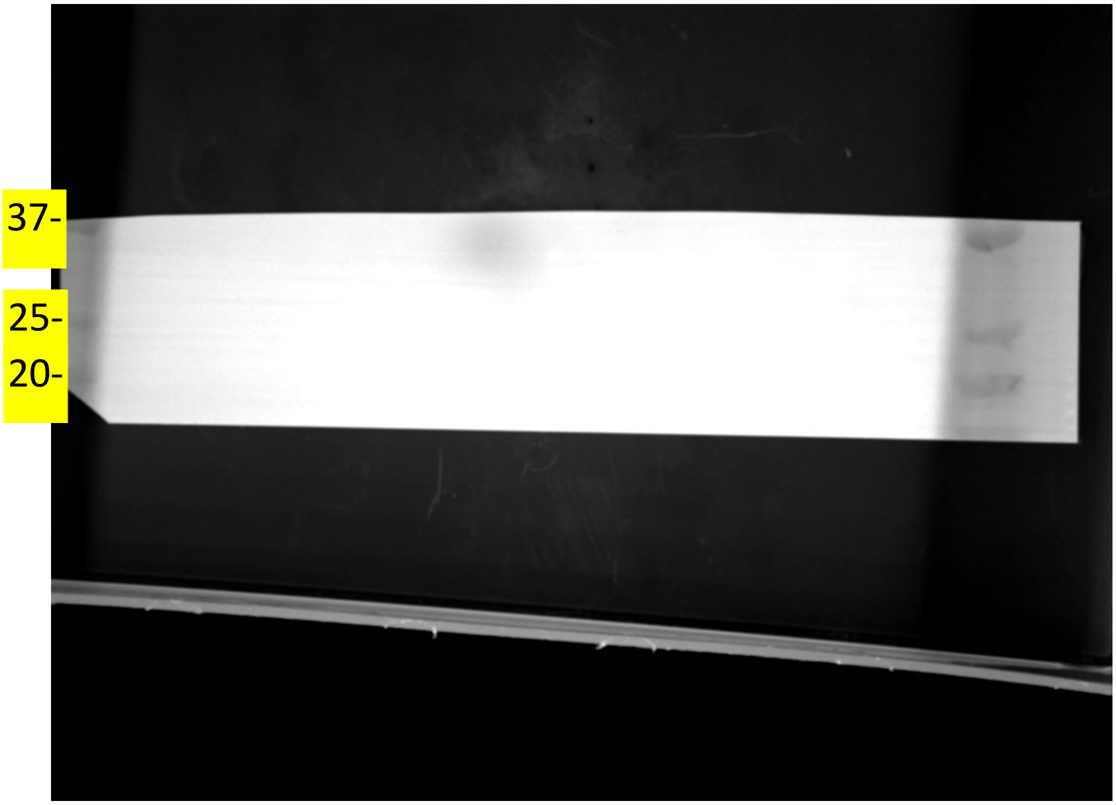

Figure 2 – E-cadherin (135 kDa)

Top: uncropped western blot with labels placed according to visible light image

Bottom: uncropped visible light image with labeled protein standards

Ladder used: Biorad Precision Plus Protein Standard, Dual color (1610374)

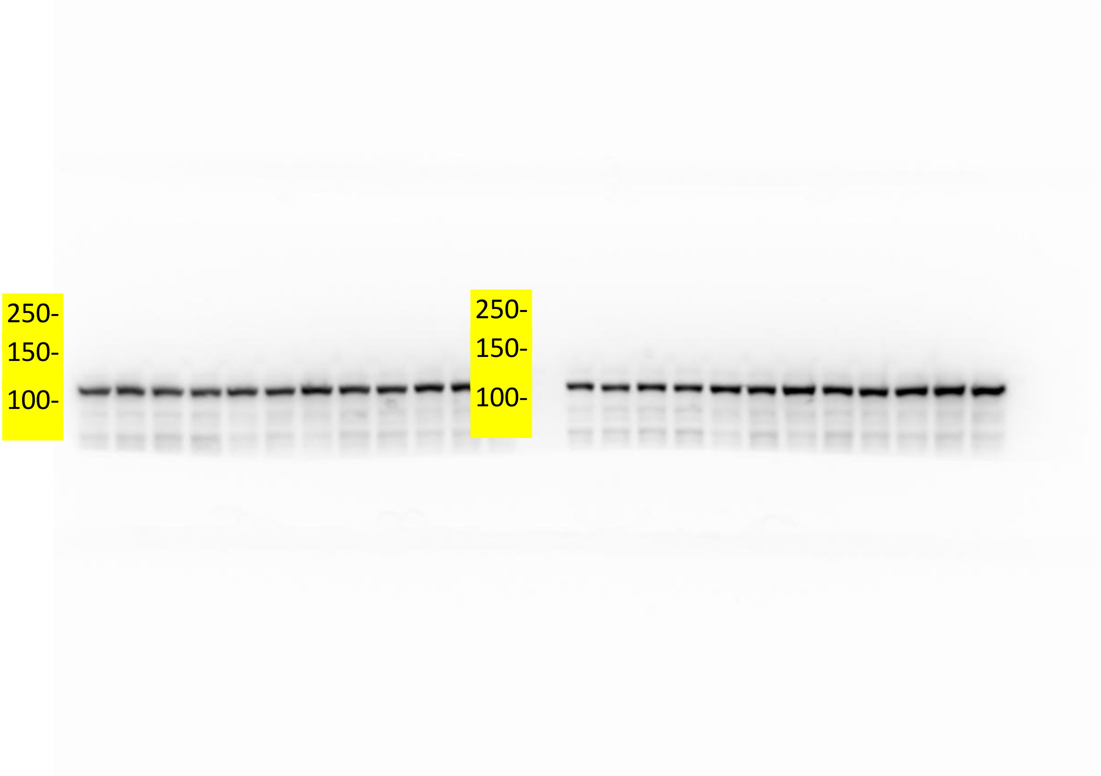

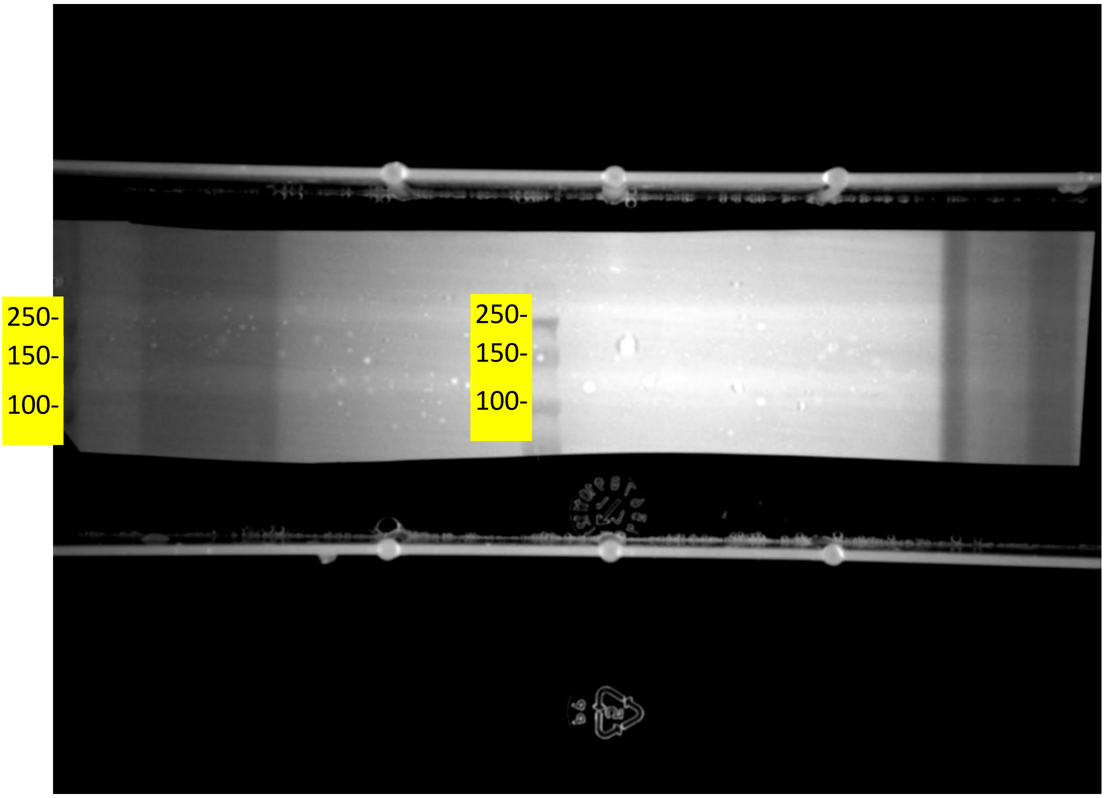

Figure 2 – Vimentin (60 kDa)

Top: uncropped western blot with labels placed according to visible light image

Bottom: uncropped visible light image with labeled protein standards

Ladder used: Biorad Precision Plus Protein Standard, Dual color (1610374)

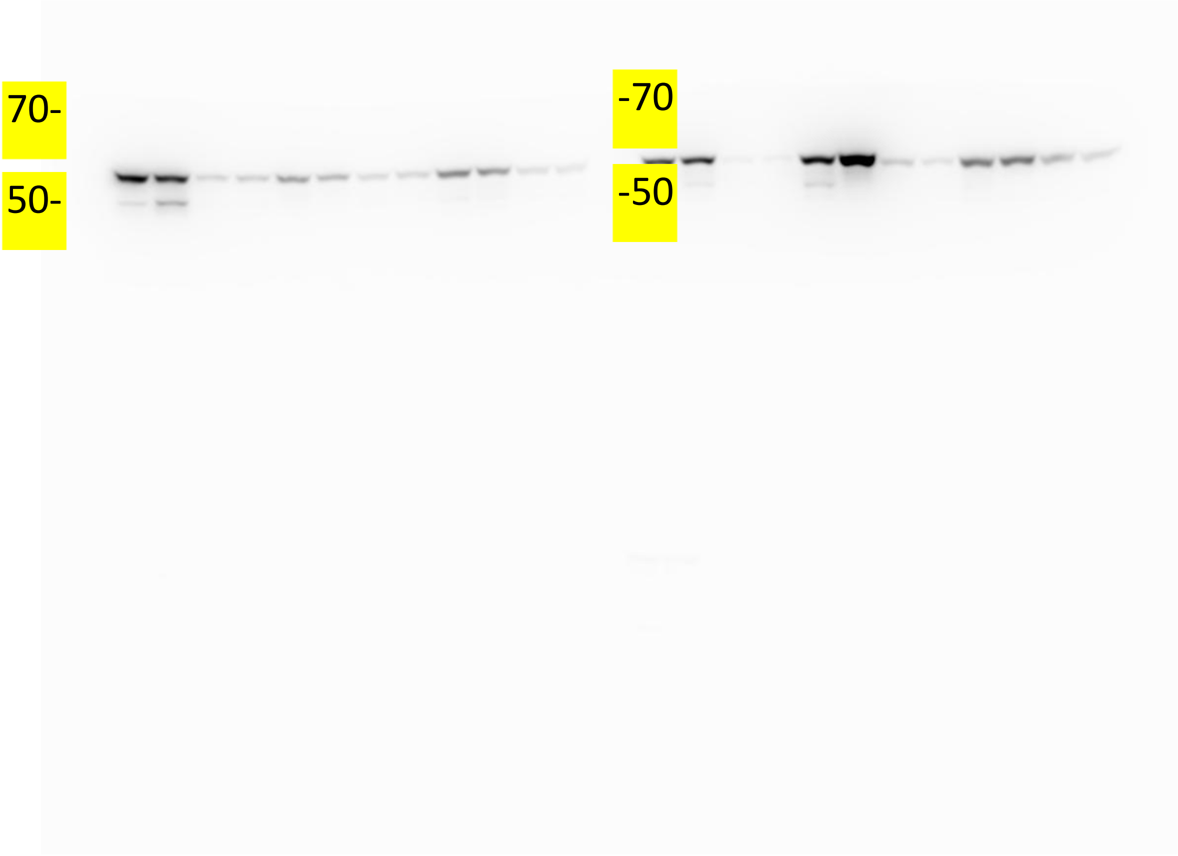

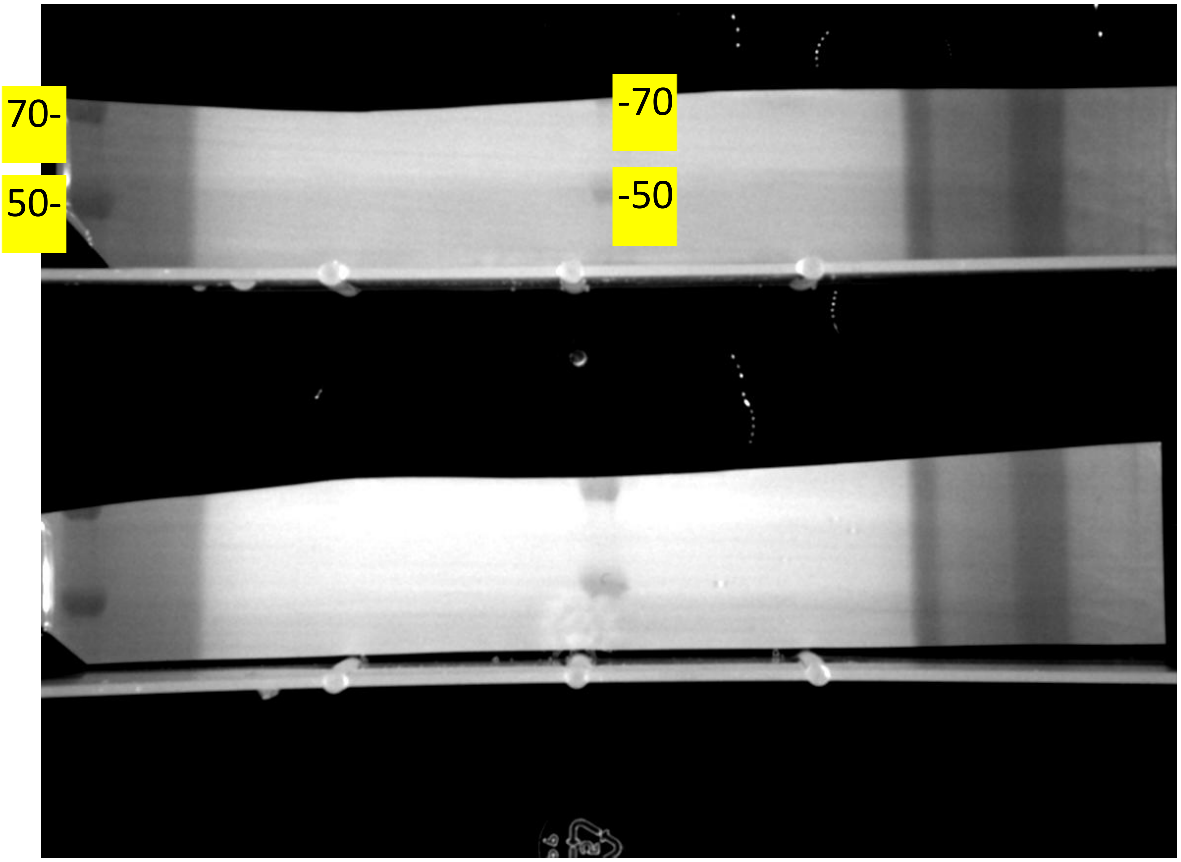

Figure 2 – Slug (30 kDa)

Top: uncropped western blot with labels placed according to visible light image

Bottom: uncropped visible light image with labeled protein standards

Ladder used: Biorad Precision Plus Protein Standard, Dual color (1610374)

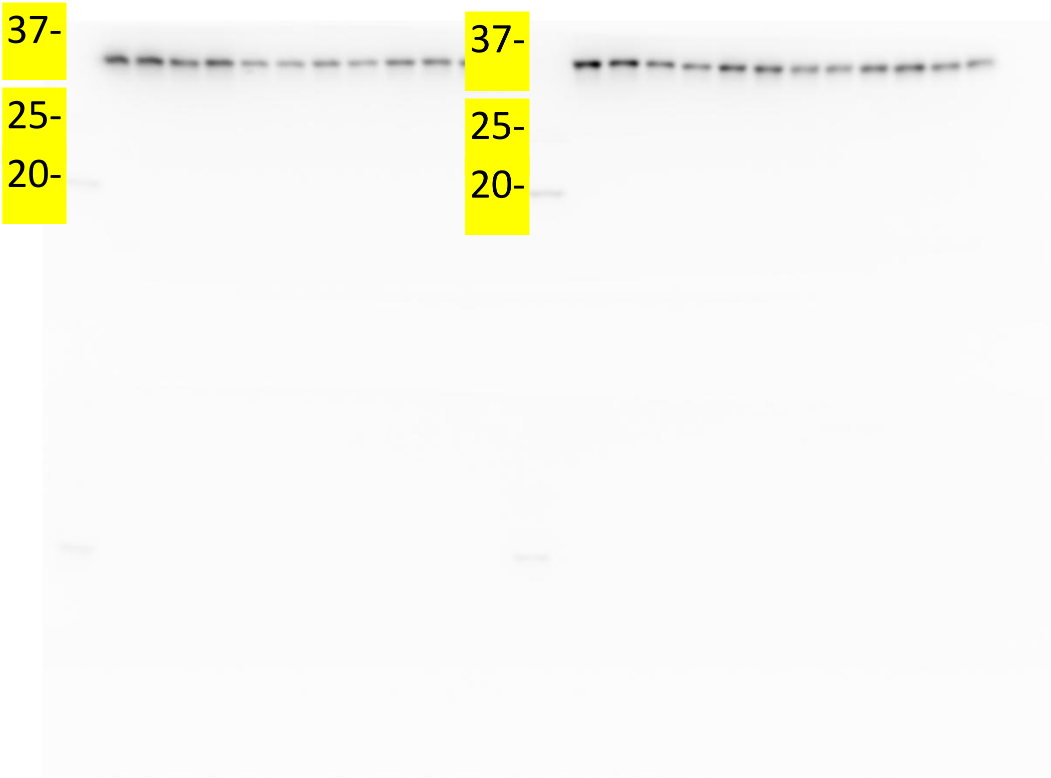

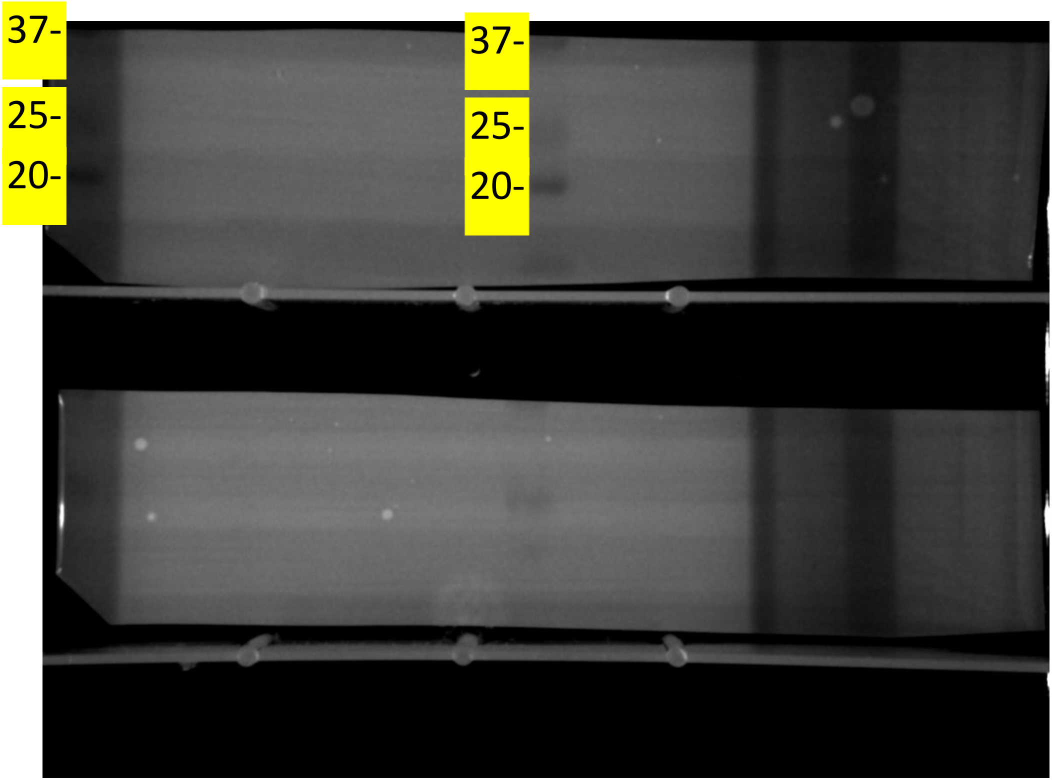

Figure 2 – GAPDH (34 kDa)

Top: uncropped western blot with labels placed according to visible light image

Bottom: uncropped visible light image with labeled protein standards

Ladder used: Biorad Precision Plus Protein Standard, Dual color (1610374)

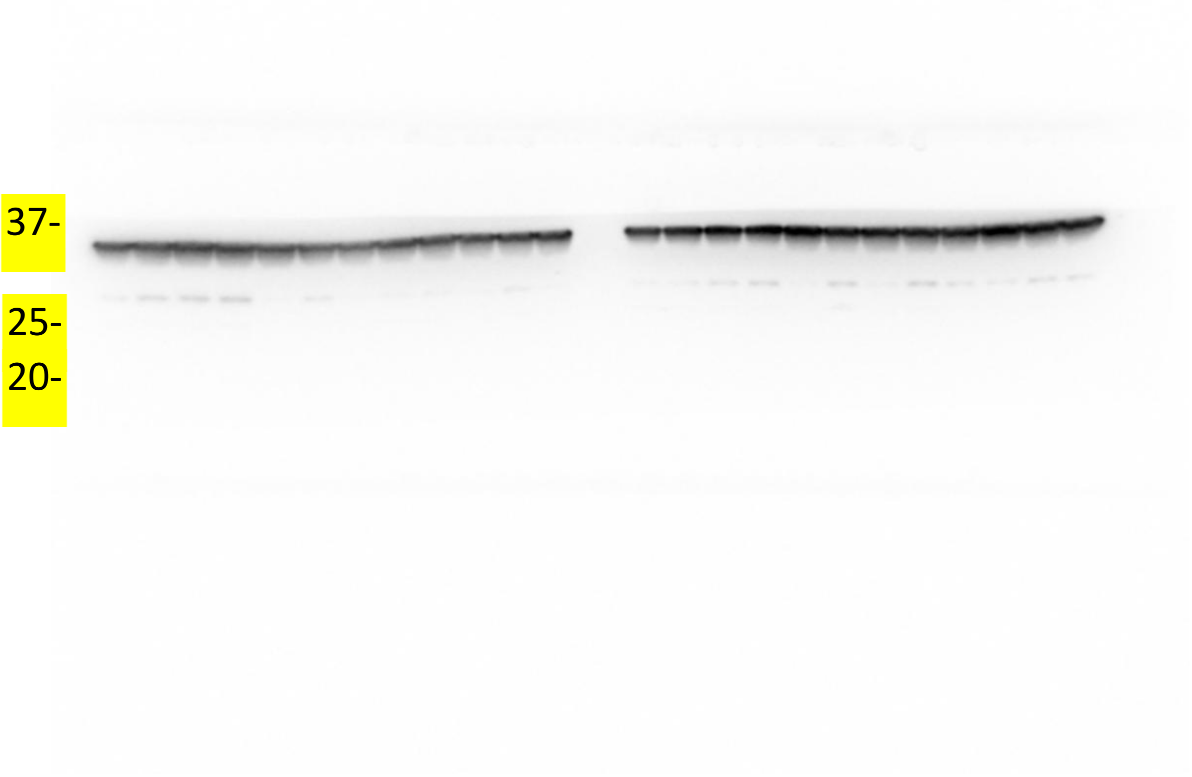

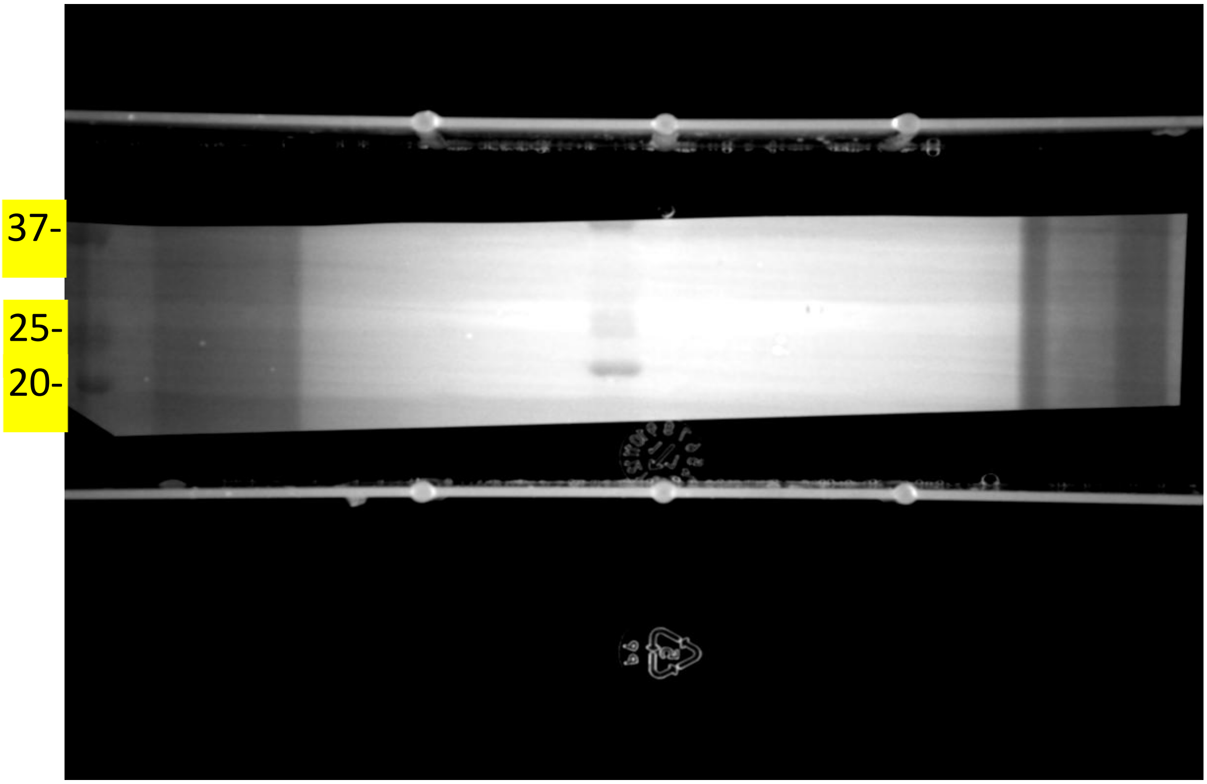

Figure 4 – E-cadherin (135 kDa)

Uncropped western blot with labels placed according to visible light image

Visible light image was not captured for this blot. The PVDF membrane was cut after transfer just below the 100 kDa marker from the visible ladder (as in previous blots for Figures 1 and 2)

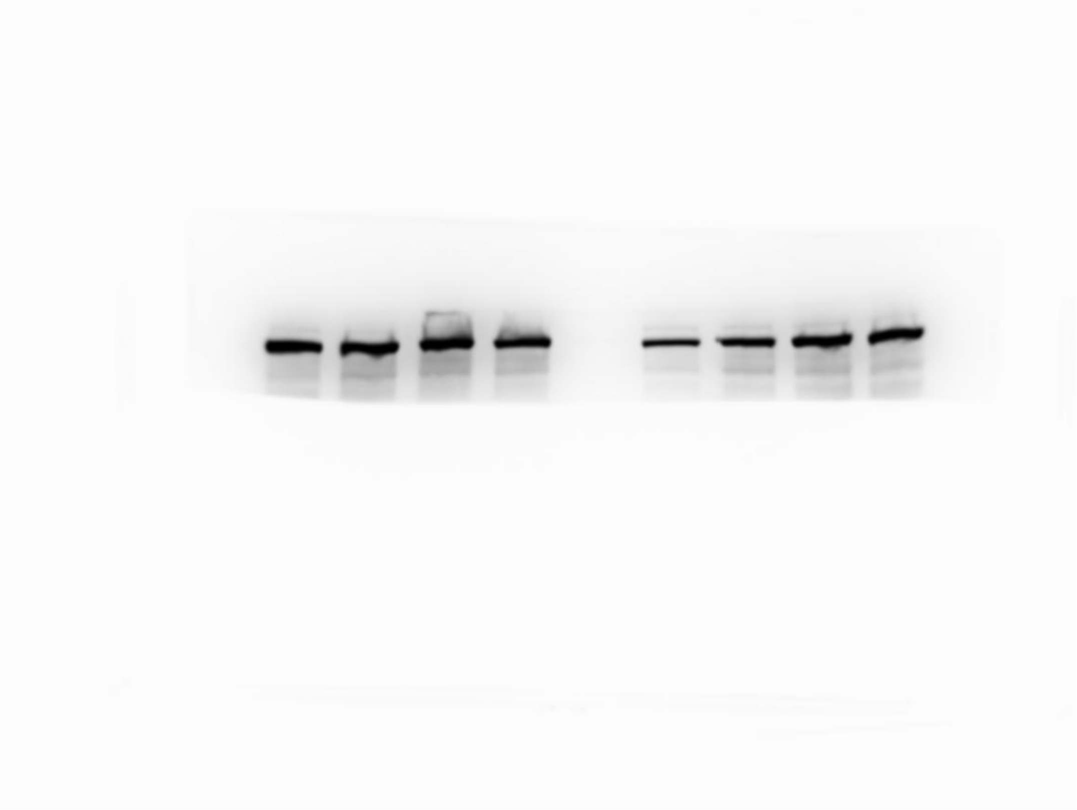

Figure 4 – Vimentin (60 kDa)

Top: uncropped western blot with labels placed according to visible light image

Bottom: uncropped visible light image with labeled protein standards

Ladder used: Biorad Precision Plus Protein Standard, Dual color (1610374)

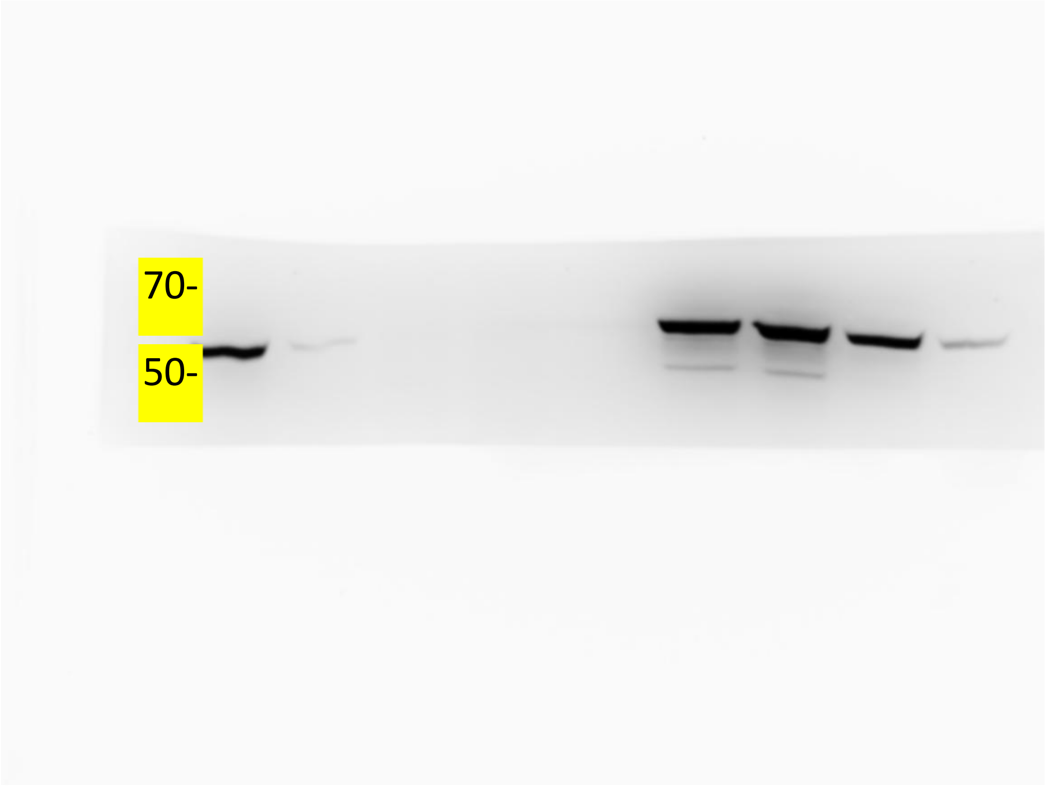

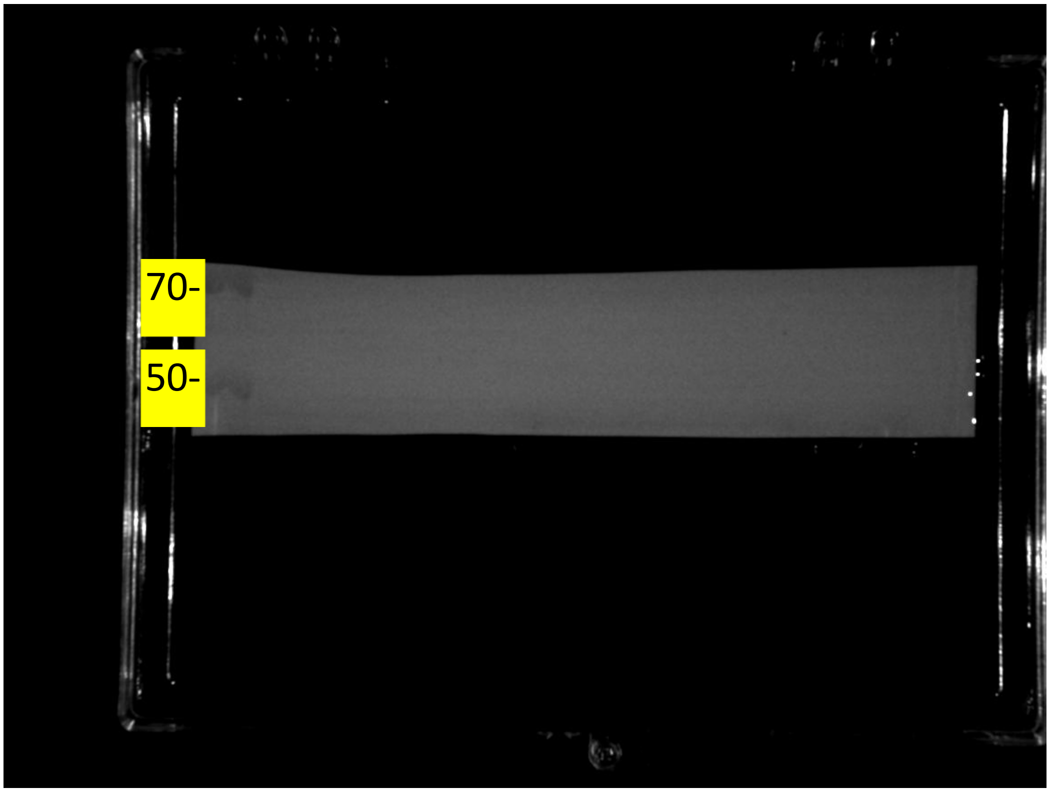

Figure 4 – Slug (30 kDa)

Top: uncropped western blot with labels placed according to visible light image

Bottom: uncropped visible light image with labeled protein standards

Ladder used: Biorad Precision Plus Protein Standard, Dual color (1610374)

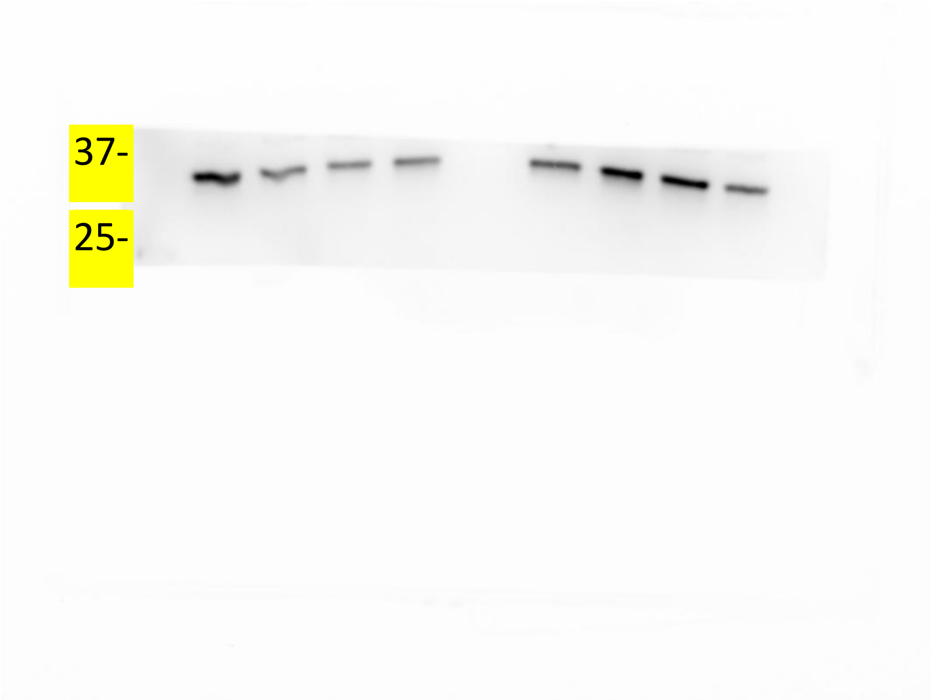

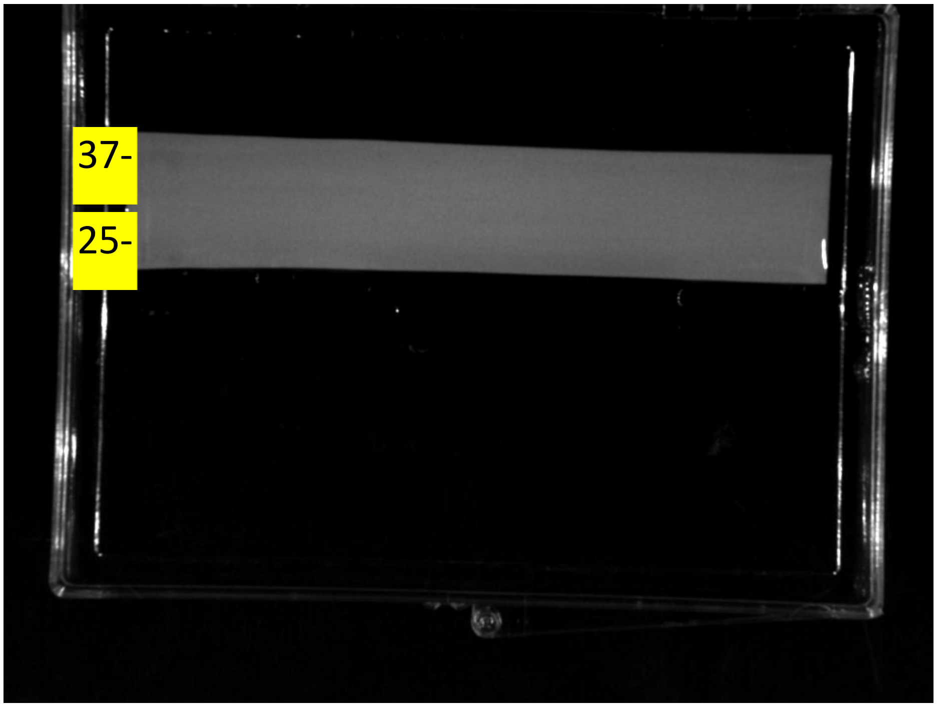

Figure 4 – GAPDH (34 kDa)

Top: uncropped western blot with labels placed according to visible light image

Bottom: uncropped visible light image with labeled protein standards

Ladder used: Biorad Precision Plus Protein Standard, Dual color (1610374)

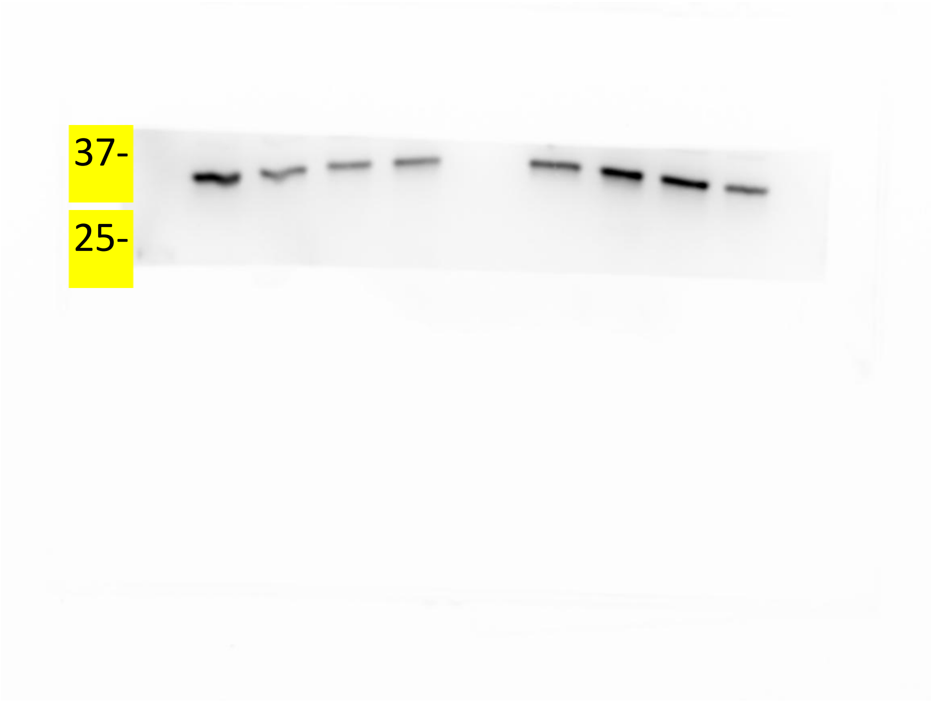

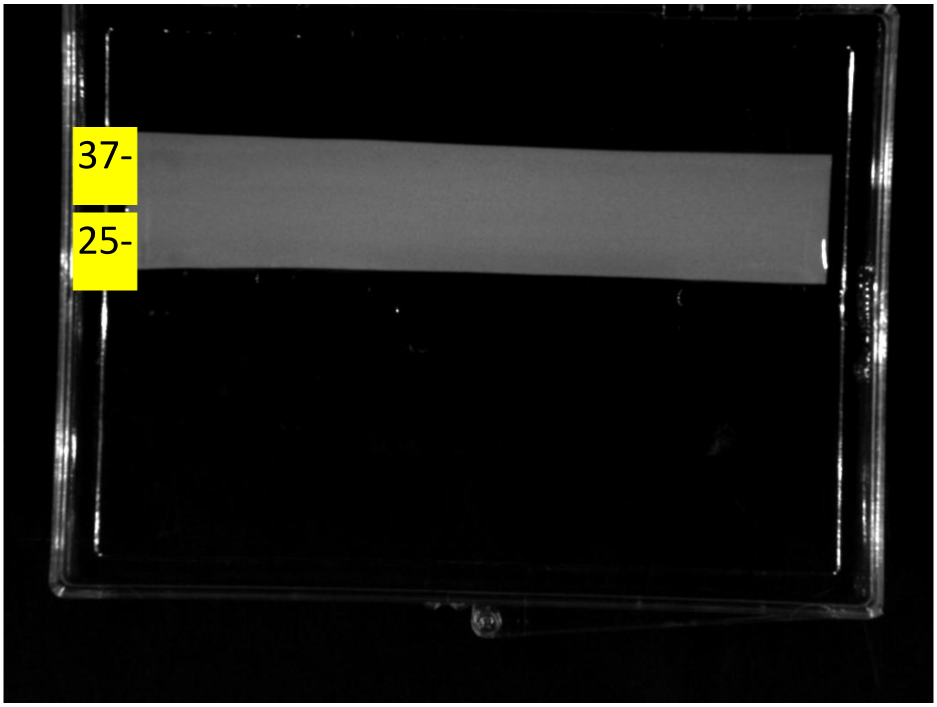

Supplement: Document S1. Figures S1–S2 and Data S1/Methods S1 [file mmc1.pdf]
